# Comparison of Instrumental Variable Methods With Continuous Exposure and Binary Outcome: A Simulation Study

**DOI:** 10.2188/jea.JE20230271

**Published:** 2025-01-05

**Authors:** Shunichiro Orihara, Atsushi Goto

**Affiliations:** 1 Department of Health Data Science, Tokyo Medical University, Tokyo, Japan; 2 Department of Public Health, School of Medicine, Yokohama City University, Yokohama, Japan

**Keywords:** inverse-variance weighted method, limited information maximum likelihood, two-stage least square, two-stage residual inclusion, weak instrument bias

## Abstract

**Background:**

Instrumental variable (IV) methods are widely employed to estimate causal effects when concerns regarding unmeasured confounders. Although comparisons among several IV methods for binary outcomes exist, comprehensive evaluations are insufficient. Therefore, in this study, we aimed to conduct a simulation with some settings for a detailed comparison of these methods, focusing on scenarios where IVs are valid and under effect homogeneity with different instrument strengths.

**Methods:**

We compared six IV methods under 32 simulation scenarios: two-stage least squares (2SLS), two-stage predictor substitutions (2SPS), two-stage residual inclusions (2SRI), limited information maximum likelihood (LIML), inverse-variance weighted methods with a linear outcome model (IVW_LI_), and inverse-variance weighted methods with a non-linear model (IVW_LL_). By comparing these methods, we examined three key estimates: the parameter estimates of the exposure variable, the causal risk ratio, and the causal risk differences.

**Results:**

Based on the results, six IV methods could be classified into three groups: 2SLS and IVW_LI_, 2SRI and 2SPS, and LIML and IVW_LL_. The first pair showed a clear bias owing to outcome model misspecification. The second pair showed a relatively good performance when strong IVs are available; however, the estimates suffered from a significant bias when only weak IVs are used. The third pair produced relatively conservative results, although they were less affected by weak IV issues.

**Conclusion:**

The findings indicate that no panacea is available for the bias associated with IV methods. We suggest using multiple IV methods: one for primary analysis and another for sensitivity analysis.

## INTRODUCTION

Instrumental variable (IV) methods are widely employed to estimate causal effects when concerns regarding unmeasured confounders.^1^ IV methods require the use of (valid) IVs that satisfy three key conditions: 1) the IVs were related to the exposure, 2) the IVs were related to the outcome only through the exposure, and 3) the IV was independent of unmeasured exposure-outcome and instrument-outcome confounders. Their strength lies in enabling users to estimate unbiased causal effects. Notably, at least one of two additional assumptions—the homogeneity assumption^2^ or monotonicity assumption^3^—is necessary to identify the causal effects using IVs. In the following discussion, we assume the former assumption.

A well-known IV method is the two-stage least squares (2SLS).^4^ In this method, the IVs are regressed on the exposure variable of interest to obtain the predictors of exposure in the first stage, and the predicted exposures are regressed on the outcome variables of interest in the second stage. The 2SLS is often preferred because of its ease of use and robustness in handling misspecifications of the first-stage model. Another commonly used IV method is the two-stage predictor substitution (2SPS),^5^ which is suitable when the outcome variables are binary, multinomial, count, or of other types. The 2SPS typically employs a generalized linear model in the second stage. Notably, the concepts of 2SLS and 2SPS can be extended to the situation where one dataset is split for first-stage estimation and the other is used for second-stage estimation. This extension of the estimation strategy is known as the inverse-variance weighted (IVW) method.^2^ Another well-known approach is the two-stage residual inclusion (2SRI),^5^ which employs a different second stage from that of 2SPS. Specifically, in 2SRI, the exposure residuals are regressed on the outcome variable of interest. The limited information maximum likelihood (LIML) method^4^^,^^6^ is another option for estimating causal effects when dealing with binary, multinomial, count, and other types of outcomes. LIML is similar to 2SPS and 2SRI, but it involves only a one-step estimation and requires specification of the distribution of unmeasured confounders. Although this assumption is strong, LIML can yield unbiased causal effects^7^^,^^8^ even in cases where 2SPS and 2SRI may produce biased causal effects^9^ except in the case of rare diseases.^10^ More details and R code of these methods are presented in eMaterial 1.

As previously mentioned, numerous IV methods exist for analyzing binary, multinomial, count, and other types of outcomes. Additionally, there is a considerable body of simulation studies comparing some of these methods; for instance, only 2SLS,^11^ 2SLS versus 2SPS versus 2SRI,^10^ and 2SPS versus 2SRI versus LIML.^7^^,^^8^ In the present study, building upon previous studies, we aim to provide a comprehensive side-by-side comparison of all of these methods, especially in situations where many IVs are used. The scenario is commonly encountered in Mendelian Randomization (MR),^2^^,^^12^^,^^13^ where genetic mutations, particularly single-nucleotide polymorphisms (SNPs), are used as IVs. However, the results of the simulation study in this manuscript are not restricted to MR; they can potentially be extended to the preference-based approach,^14^ another IV method commonly used in epidemiology, biometrics, and other related fields.

MR can be implemented in one-sample and two-sample designs. This manuscript focuses on the one-sample MR, which uses only one dataset for data analysis and follows a process similar to ordinary data analysis. Conversely, the two-sample MR uses two datasets or splits one dataset to address different objectives. One advantage of one-sample MR is that the model analysis, including baseline covariates, such as age and sex, is easy to implement. If the covariates are important risk factors, including them can increase the efficiency of the causal effect estimate. Another advantage is that subgroup analysis is easy to perform. More information on MR can be found in book^2^ and review papers.^12^^,^^13^

## METHODS

An artificial simulation dataset was used for this study. The dataset was designed to investigate the causal effects of a continuous exposure on a binary outcome, with many weak IVs applicable. This scenario is particularly relevant for MR; for instance, assessing the impact of body mass index (BMI; exposure) on the incidence of diabetes (outcome) using SNPs (ie, IVs) that capture the genetic variation associated with BMI. However, as mentioned in the introduction, the simulation experiments are not confined to MR; the settings and results can potentially be extended to other observational study situations, such as database research using preference-based IVs.

### Data-generating mechanisms (DGM)

The simulation dataset was generated according to the following probability distribution, primarily referencing Gkatzionis et al.^15^ The sample size *n* was 100,000, and the simulation was iterated 200 times. First, assuming the existence of 500 IVs, each IV *Z_k_* was generated from a binomial distribution $Z_{k}∼Bin(2,q_{k})$ , where *q_k_* was generated from a uniform distribution $q_{k}∼Unif(0.1,0.9)$ . For MR, the binomial distribution can be considered as the distribution of genetic variations, where *Z_k_* = 0 corresponds to no variation (ie, wild type), *Z_k_* = 1 corresponds to a single variation, and *Z_k_* = 2 corresponds to double variations. Next, we assumed the presence of two unmeasured confounders, denoted as $(V,U{)}^{⊤}$ , which showed a continuous distribution with a correlation coefficient *ρ* and were independent from the IVs (*Z_k_*s) defined above. Note that the bivariate distribution is not always considered in the context of unmeasured confounders. However, as explained later, this setting is necessary for applying LIML. Also, the other IV methods considered in this manuscript can be applied. In addition, a single measured confounder, denotes age, had the following distribution: $X∼N(55,7^{2})$ . Finally, we introduced models for exposure and outcomes. The exposure followed the following linear model:

$$T=20+\sum_{k=1}^{25}Z_{k}\alpha_{k}+\sum_{k=26}^{500}Z_{k}\alpha_{k}^{\prime }+0.2X+V.$$ (1)

Here, $\alpha_{k}∼|N(0.02,0.07^{2})|$ and $\alpha_{k}^{\prime }∼|N(0.001,0.01^{2})|$ . *α_k_* indicated a strong relationship between the IVs and the exposure, whereas $\alpha_{k}^{\prime }$ indicated a weak relationship. We obtained 25 strong and 475 weak IVs. Considering this formulation, the mean of the exposure variable was approximately 37.63 (standard deviation, 2.70). The outcomes were generated from the following linear model:

Y=1{−7.5+0.1T+0.05X+U},

where **1**{*A*} denotes the indicator function, which equals 1 when *A* is true, and 0 otherwise. Since *U* has the standard normal distribution, the outcome follows a probit model. Considering this formulation, the incidence rate of the outcome is approximately 15%, indicating that it is a rare event. The risk ratio $\frac{Pr(Y=1|T=t_{1},X=55)}{Pr(Y=1|T=t_{0},X=55)}$ is assumed to increase by approximately 4.15 with a 10-unit increase in exposure (ie, *t*_1_ − *t*_0_ = 10). Note that under the DGM, three IV conditions were met^1^: 1) the IVs were related to the exposure, 2) the IVs were related to the outcome only through the exposure, and 3) the IV was independent of unmeasured confounders. Additionally, the homogeneity assumption is maintained in the outcome model, as there are no interaction terms between the exposure and unmeasured confounders.

Considering the aforementioned DGM, 32 simulation settings with different patterns were explored. Specifically, we examined three key options.

**Figure 1.**
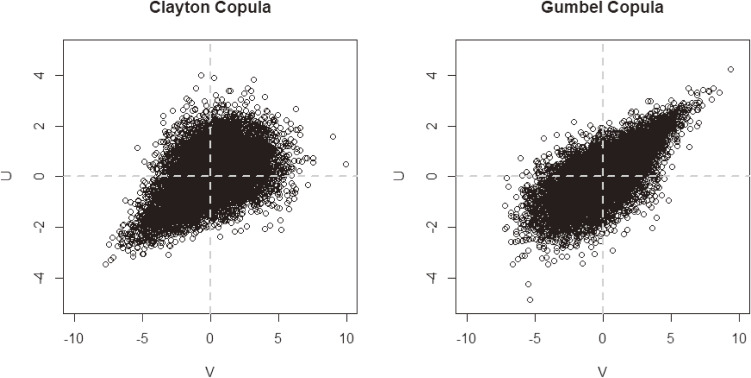
Scatter plot of Clayton and Gumbel copula (unmeasured confounders) in a simulation iteration

• Use of 25 strong IVs and 25 weak IVs/only 50 weak IVs
• Inclusion/exclusion of a measured confounder (*X*)
• Assumptions of various unmeasured confounder ( (V,U) ) distribution options constructed based on copulas^16^ (see Figure 1):
  ➢ Symmetry and light-tailed joint distribution (ie, a bivariate normal distribution) with a correlation coefficient of *ρ* = 0.5/−0.5
  ➢ Symmetry, but joint distribution with heavy-tailed marginal distributions (ie, normal distributions with t-copula) with a correlation coefficient of *ρ* = 0.5/−0.5
  ➢ Asymmetry, with light-tailed marginal distributions (ie, normal distributions with Clayton/Gumbel copula) with a correlation coefficient of *ρ* = 0.5
  ➢ Symmetry, but marginal distributions with a heavy-tailed distribution (ie, t-distributions with normal copula) with a correlation coefficient of *ρ* = 0.5/−0.5

Note that in the first option, “Use of 25 strong IVs and 25 weak IVs/only 50 weak IVs” refers to the utilization of IVs to implement IV methods explained soon after. Consequently, we could not detect a valid exposure model exactly in all cases (ie, estimation models are different from the true construction (1)).

### Estimating methods

In the simulation analysis, we compared six methods—2SLS, 2SPS, 2SRI, LIML—and two IVW. For the 2SLS and one IVW methods, a linear model is used in the second stage. This IVW method is termed “IVW under linear model (IVW_LI_).” For 2SPS, 2SRI, both probit and log link functions were used in the second stage. The probit link application is denoted as “2SRI_PR_” and “2SPS_PR_”, while the log link application is denoted as “2SRI_LL_” and “2SPS_LL_”. Similarly, the other IVW method is referred to as “IVW under non-linear model (IVW_LL_).” Note that for all the methods mentioned above, the first-stage model was a linear model.

For the LIML, the probit outcome and linear exposure models were followed; however, the estimation methods differed. The joint likelihood of the outcome and exposure variables was used to estimate the parameters of interest. The joint likelihood was derived based on the assumption that unmeasured confounders follow a bivariate normal distribution. Therefore, except for the “Symmetry and light-tailed distribution” situation, the assumed distributions of the unmeasured confounders were misspecified.

For the two IVW methods, the first and second steps were implemented using separate datasets of 50,000 subjects to simulate a two-sample MR setting in a pseudo manner. In the simulation experiments, the first dataset is used for estimating the IV-exposure relationship, and the second dataset is used for estimating the IV-outcome relationship in each iteration. Further, the datasets are replaced, and the process is repeated. Therefore, two causal effect estimators are obtained in each iteration. The final estimator is obtained by averaging these two estimators. Two-sample MR methods are widely recognized to yield more conservative results (ie, they do not tend to produce type 1 errors) than one-sample MR methods.^12^ This is considered an advantage of the two-sample MR method.

### Performance metrics

To evaluate these methods, we primarily used two metrics: mean and empirical standard error (ESE) for the estimated parameters of interest (ie, the exposure variable) called “beta coefficient” and the estimated causal risk ratio (CRR). For the 2SLS, 2SPS, 2SRI, and LIML methods, when the model included the measured confounders, we conducted a G-formula based estimation^17^ to estimate the CRR.

• For 2SPS_PR_/2SRI_PR_/LIML methods, $\hat{p}(t^{\prime })=\frac{1}{n}\sum_{i=1}^{n}\Phi ({\hat{\alpha }}_{0}+t^{\prime }{\hat{\alpha }}_{t}+x_{i}^{⊤}{\hat{\alpha }}_{x})$
• For 2SPS_LL_/2SRI_LL_ methods, $\hat{p}(t^{\prime })=\frac{1}{n}\sum_{i=1}^{n}exp({\hat{\alpha }}_{0}+t^{\prime }{\hat{\alpha }}_{t}+x_{i}^{⊤}{\hat{\alpha }}_{x})$
• For the 2SLS method, $\hat{p}(t^{\prime })=\frac{1}{n}\sum_{i=1}^{n}({\hat{\alpha }}_{0}+t^{\prime }{\hat{\alpha }}_{t}+x_{i}^{⊤}{\hat{\alpha }}_{x})$
• For the IVW_LL_ methods, $\hat{p}(t^{\prime })=exp({\hat{\alpha }}_{0}+t^{\prime }{\hat{\alpha }}_{t})$
• For the IVW_LI_ method, $\hat{p}(t^{\prime })={\hat{\alpha }}_{0}+t^{\prime }{\hat{\alpha }}_{t}$

Here, *Φ* is the distribution function of the standard normal distribution, *x_i_* is a vector of covariates, and ${\hat{\alpha }}_{j}$ represents the estimated parameters obtained using each method. Therefore, the CRR was calculated as $\hat{p}(t_{2})/\hat{p}(t_{1})$ when the amount of risk increases/decreases when the exposure variable increases from *t*_1_ to *t*_2_. When the model did not include the measured confounders, we used the corresponding mapping based on probability distribution.

• For 2SPS_PR_/2SRI_PR_/LIML methods, $\hat{p}(t^{\prime })=\Phi ({\hat{\alpha }}_{0}+t^{\prime }{\hat{\alpha }}_{t})$
• For 2SPS_LL_/2SRI_LL_/IVW_LL_ methods, $\hat{p}(t^{\prime })=exp({\hat{\alpha }}_{0}+t^{\prime }{\hat{\alpha }}_{t})$
• For the 2SLS/IVW_LI_ method, $\hat{p}(t^{\prime })={\hat{\alpha }}_{0}+t^{\prime }{\hat{\alpha }}_{t}$

For more details on the estimation process, refer to Angrist et al^18^ or Burgess et al.^19^ Further explanations of the other metrics are provided in the subsequent section, as necessary.

In addition to the two metrics, the causal risk differences (CRD), defined as $\hat{p}(t_{2})-\hat{p}(t_{1})$ when the amount of risk increases/decreases when the exposure variable increases from *t*_1_ to *t*_2_, is another important parameter for estimating the causal effect for the dichotomous outcomes.^2^^,^^20^ Note that $\hat{p}(t)$ is estimated using the same method as the CRR is, as explained in the previous paragraph. We summarize the results for CRD and detail the differences from CRR in eMaterial 2 and eTable 1, eTable 2, eTable 3, eTable 4, eTable 5, eTable 6, eTable 7, eTable 8, eTable 9, eTable 10, eTable 11, and eTable 12.

## RESULTS

The estimated parameters of interest (ie, the exposure variables) are summarized in Table 1 and Table 2. The estimated CRR under scenarios where BMI increased from 30.5 to 37.0 and from 41.0 to 47.5 are summarized in Table 3, Table 4, Table 5, and Table 6, respectively. The results of CRR under scenarios where BMI increased from 18.5 to 25.0 and from 53.0 to 59.5 are stored in the supplemental material. Overall, the CRD results show the same tendency as the CRR; almost all consistent results are obtained with both CRD and CRR.

**Table 1.** Summary of beta coefficient with age (measured confounder) included in the outcome model

Estimators	Simulation Settings							

	Using 25 Strong IVs and 25 Weak IVs							

	Bivariate Normal Distribution		Marginal Normal Distributions				Marginal t-distributions	
	
			With t-copula		With Clayton Copula	With Gumbel Copula	With Normal Copula	

	*ρ*^a^ = 0.5	*ρ*^a^ = −0.5	*ρ*^a^ = 0.5	*ρ*^a^ = −0.5	*ρ*^a^ = 0.5	*ρ*^a^ = 0.5	*ρ*^a^ = 0.5	*ρ*^a^ = −0.5
2SRI_PR_	0.142 (0.023)	0.096 (0.023)	0.132 (0.023)	0.090 (0.023)	0.202 (0.030)	0.121 (0.022)	0.124 (0.030)	0.099 (0.027)
2SRI_LL_	0.147 (0.026)	0.130 (0.032)	0.142 (0.026)	0.124 (0.031)	0.145 (0.025)	0.139 (0.026)	0.139 (0.025)	0.126 (0.036)
LIML	0.091 (0.179)	0.081 (0.097)	0.080 (0.179)	0.078 (0.144)	0.357 (0.304)	0.101 (0.025)	0.098 (0.304)	0.341 (0.054)
2SLS	0.026 (0.004)	0.018 (0.004)	0.480 (0.004)	0.017 (0.004)	0.027 (0.004)	0.025 (0.005)	0.024 (0.004)	0.019 (0.005)
2SPS_PR_	0.109 (0.018)	0.091 (0.022)	0.104 (0.018)	0.087 (0.022)	0.109 (0.017)	0.105 (0.019)	0.102 (0.017)	0.089 (0.025)
2SPS_LL_	0.146 (0.025)	0.130 (0.032)	0.141 (0.025)	0.123 (0.031)	0.147 (0.023)	0.139 (0.026)	0.139 (0.023)	0.126 (0.036)
IVW_LI_	0.019 (0.004)	0.018 (0.004)	0.017 (0.004)	0.017 (0.004)	0.018 (0.004)	0.019 (0.005)	0.020 (0.004)	0.020 (0.005)
IVW_LL_	0.104 (0.025)	0.134 (0.030)	0.099 (0.025)	0.126 (0.030)	0.096 (0.024)	0.104 (0.027)	0.120 (0.024)	0.134 (0.035)



Estimators	Simulation Settings							

	Using Only 50 Weak IVs							

	Bivariate Normal Distribution		Marginal Normal Distributions				Marginal t-distributions	
	
			With t-copula		With Clayton Copula	With Gumbel Copula	With Normal Copula	

	*ρ*^a^ = 0.5	*ρ*^a^ = −0.5	*ρ*^a^ = 0.5	*ρ*^a^ = −0.5	*ρ*^a^ = 0.5	*ρ*^a^ = 0.5	*ρ*^a^ = 0.5	*ρ*^a^ = −0.5

2SRI_PR_	0.271 (0.056)	−0.038 (0.053)	0.242 (0.056)	−0.033 (0.064)	0.461 (0.072)	0.199 (0.049)	0.229 (0.072)	−0.030 (0.080)
2SRI_LL_	0.277 (0.063)	−0.051 (0.074)	0.267 (0.063)	−0.050 (0.088)	0.336 (0.081)	0.226 (0.059)	0.244 (0.081)	−0.035 (0.105)
LIML	0.128 (0.161)	0.047 (0.132)	0.136 (0.161)	0.052 (0.133)	0.622 (0.174)	0.232 (0.120)	0.127 (0.174)	0.340 (0.146)
2SLS	0.050 (0.011)	−0.007 (0.010)	0.401 (0.011)	−0.007 (0.012)	0.062 (0.011)	0.040 (0.010)	0.043 (0.011)	−0.006 (0.016)
2SPS_PR_	0.208 (0.047)	−0.038 (0.050)	0.197 (0.047)	−0.035 (0.062)	0.253 (0.043)	0.169 (0.043)	0.186 (0.043)	−0.029 (0.076)
2SPS_LL_	0.280 (0.062)	−0.053 (0.071)	0.266 (0.062)	−0.050 (0.088)	0.337 (0.058)	0.227 (0.059)	0.253 (0.058)	−0.039 (0.107)
IVW_LI_	0.007 (0.011)	0.006 (0.008)	0.005 (0.011)	0.004 (0.009)	0.005 (0.013)	0.005 (0.011)	0.009 (0.013)	0.011 (0.015)
IVW_LL_	0.036 (0.063)	0.041 (0.057)	0.029 (0.063)	0.031 (0.067)	0.025 (0.069)	0.031 (0.060)	0.055 (0.069)	0.071 (0.096)

IV, instrumental variable; IVW_LI_, inverse-variance weighted estimator with linear construction; IVW_LL_, inverse-variance weighted estimator with non-linear construction; LIML, limited information maximum likelihood; 2SLS, two-stage least square; 2SPS_PR_, two-stage predictor substitution with probit link; 2SPS_LL_, two-stage predictor substitution with log link; 2SRI_PR_, two-stage residual inclusion with probit link; 2SRI_LL_, two-stage residual inclusion with log link.

The sample size *n* is 100,000, and the simulation is iterated 200 times. The true value is 0.1. The mean and empirical standard error (ESE) represented as “mean (ESE).” For the IVW methods, the datasets were split into two subsets, each containing 50,000 samples.

^a^*ρ* is a correlation coefficient between two unmeasured confounders.

**Table 2.** Summary of beta coefficient without age (measured confounder) in the outcome model

Estimators	Simulation Settings							

	Using 25 Strong IVs and 25 Weak IVs							

	Bivariate Normal Distribution		Marginal Normal Distributions				Marginal t-distributions	
	
			With t-copula		With Clayton Copula	With Gumbel Copula	With Normal Copula	

	*ρ*^a^ = 0.5	*ρ*^a^ = −0.5	*ρ*^a^ = 0.5	*ρ*^a^ = −0.5	*ρ*^a^ = 0.5	*ρ*^a^ = 0.5	*ρ*^a^ = 0.5	*ρ*^a^ = −0.5
2SRI_PR_	0.150 (0.022)	0.089 (0.020)	0.140 (0.022)	0.084 (0.021)	0.210 (0.029)	0.129 (0.022)	0.129 (0.029)	0.089 (0.023)
2SRI_LL_	0.156 (0.025)	0.144 (0.032)	0.151 (0.025)	0.135 (0.033)	0.153 (0.024)	0.150 (0.026)	0.148 (0.024)	0.138 (0.036)
LIML	0.224 (0.211)	0.198 (0.126)	0.256 (0.211)	0.133 (0.104)	0.200 (0.201)	0.132 (0.143)	0.080 (0.201)	0.174 (0.229)
2SLS	0.028 (0.004)	0.019 (0.004)	0.130 (0.004)	0.018 (0.004)	0.028 (0.004)	0.027 (0.005)	0.025 (0.004)	0.021 (0.005)
2SPS_PR_	0.106 (0.016)	0.089 (0.020)	0.102 (0.016)	0.084 (0.021)	0.107 (0.016)	0.103 (0.018)	0.100 (0.016)	0.089 (0.023)
2SPS_LL_	0.155 (0.023)	0.144 (0.032)	0.150 (0.023)	0.135 (0.033)	0.154 (0.023)	0.150 (0.026)	0.149 (0.023)	0.138 (0.036)
IVW_LI_	0.017 (0.004)	0.017 (0.004)	0.016 (0.004)	0.016 (0.004)	0.016 (0.004)	0.017 (0.005)	0.019 (0.004)	0.019 (0.005)
IVW_LL_	0.098 (0.023)	0.128 (0.030)	0.092 (0.023)	0.117 (0.031)	0.090 (0.024)	0.099 (0.027)	0.113 (0.024)	0.126 (0.034)



Estimators	Simulation Settings							

	Using Only 50 Weak IVs							

	Bivariate Normal Distribution		Marginal Normal Distributions				Marginal t-distributions	
	
			With t-copula		With Clayton Copula	With Gumbel Copula	With Normal Copula	

	*ρ*^a^ = 0.5	*ρ*^a^ = −0.5	*ρ*^a^ = 0.5	*ρ*^a^ = −0.5	*ρ*^a^ = 0.5	*ρ*^a^ = 0.5	*ρ*^a^ = 0.5	*ρ*^a^ = −0.5

2SRI_PR_	0.297 (0.051)	0.034 (0.043)	0.272 (0.051)	0.035 (0.052)	0.466 (0.067)	0.234 (0.047)	0.262 (0.067)	0.068 (0.063)
2SRI_LL_	0.303 (0.057)	0.054 (0.069)	0.296 (0.057)	0.057 (0.084)	0.343 (0.074)	0.269 (0.058)	0.297 (0.074)	0.106 (0.097)
LIML	0.130 (0.172)	0.190 (0.140)	0.129 (0.172)	0.202 (0.115)	0.342 (0.220)	0.204 (0.191)	0.014 (0.220)	0.100 (0.290)
2SLS	0.054 (0.010)	0.007 (0.009)	0.430 (0.010)	0.008 (0.011)	0.064 (0.010)	0.048 (0.010)	0.051 (0.010)	0.016 (0.015)
2SPS_PR_	0.209 (0.038)	0.034 (0.043)	0.201 (0.038)	0.035 (0.052)	0.239 (0.040)	0.183 (0.039)	0.202 (0.040)	0.068 (0.062)
2SPS_LL_	0.307 (0.056)	0.055 (0.069)	0.297 (0.056)	0.057 (0.084)	0.346 (0.057)	0.268 (0.057)	0.300 (0.057)	0.106 (0.097)
IVW_LI_	0.005 (0.009)	0.005 (0.008)	0.003 (0.009)	0.003 (0.008)	0.002 (0.012)	0.004 (0.011)	0.006 (0.012)	0.006 (0.012)
IVW_LL_	0.028 (0.053)	0.033 (0.056)	0.019 (0.053)	0.020 (0.058)	0.012 (0.063)	0.024 (0.060)	0.033 (0.063)	0.042 (0.077)

IV, instrumental variable; IVW_LI_, inverse-variance weighted estimator with linear construction; IVW_LL_, inverse-variance weighted estimator with non-linear construction; LIML, limited information maximum likelihood; 2SLS, two-stage least square; 2SPS_PR_, two-stage predictor substitution with probit link; 2SPS_LL_, two-stage predictor substitution with log link; 2SRI_PR_, two-stage residual inclusion with probit link; 2SRI_LL_, two-stage residual inclusion with log link.

The sample size *n* is 100,000, and the simulation is iterated 200 times. The true value is 0.1. The mean and empirical standard error (ESE) represented as “mean (ESE).” For the IVW methods, the datasets were split into two subsets, each containing 50,000 samples.

^a^*ρ* is a correlation coefficient between two unmeasured confounders.

**Table 3.** Summary of estimated causal risk ratio when the BMI increases from 30.5 to 37.0 with age (measured confounder) included in the outcome model

Estimators	Simulation Settings							

	Using 25 Strong IVs and 25 Weak IVs							

	Bivariate Normal Distribution		Marginal Normal Distributions				Marginal t-distributions	
	
			With t-copula		With Clayton Copula	With Gumbel Copula	With Normal Copula	

	*ρ*^a^ = 0.5	*ρ*^a^ = −0.5	*ρ*^a^ = 0.5	*ρ*^a^ = −0.5	*ρ*^a^ = 0.5	*ρ*^a^ = 0.5	*ρ*^a^ = 0.5	*ρ*^a^ = −0.5
2SRI_PR_	4.045 (0.008)	2.416 (0.003)	3.661 (0.007)	2.321 (0.003)	4.757 (0.019)	3.432 (0.004)	3.147 (0.006)	2.419 (0.003)
2SRI_LL_	6.675 (0.038)	4.149 (0.017)	6.120 (0.030)	3.910 (0.015)	5.272 (0.026)	6.467 (0.025)	5.157 (0.024)	3.901 (0.019)
LIML	1.070 (0.001)	2.037 (0.003)	1.105 (0.001)	1.739 (0.002)	8.123 (0.022)	2.839 (0.002)	2.738 (0.003)	110.526 (0.669)
2SLS	2.498 (372.366)	−10.712 (>999)	5.209 (1109.217)	1.707 (224.728)	2.273 (661.464)	−19.533 (>999)	4.320 (905.831)	−2.613 (>999)
2SPS_PR_	3.138 (0.002)	2.641 (0.003)	2.976 (0.002)	2.497 (0.002)	3.150 (0.002)	2.948 (0.002)	2.878 (0.002)	2.526 (0.002)
2SPS_LL_	2.561 (0.001)	2.276 (0.001)	2.474 (0.001)	2.183 (0.001)	2.569 (0.000)	2.443 (0.001)	2.418 (0.001)	2.207 (0.001)
IVW_LI_	1.213 (0.000)	1.213 (0.000)	1.213 (0.000)	1.213 (0.000)	1.213 (0.000)	1.213 (0.000)	1.213 (0.000)	1.213 (0.000)
IVW_LL_	1.993 (0.320)	2.441 (0.484)	1.927 (0.317)	2.317 (0.481)	1.890 (0.288)	1.998 (0.355)	2.225 (0.464)	2.449 (0.562)



Estimators	Simulation Settings							

	Using Only 50 Weak IVs							

	Bivariate Normal Distribution		Marginal Normal Distributions				Marginal t-distributions	
	
			With t-copula		With Clayton Copula	With Gumbel Copula	With Normal Copula	

	*ρ*^a^ = 0.5	*ρ*^a^ = −0.5	*ρ*^a^ = 0.5	*ρ*^a^ = −0.5	*ρ*^a^ = 0.5	*ρ*^a^ = 0.5	*ρ*^a^ = 0.5	*ρ*^a^ = −0.5

2SRI_PR_	25.554 (0.164)	0.706 (0.001)	16.224 (0.065)	0.721 (0.001)	178.566 (6.912)	9.491 (0.021)	8.022 (0.036)	0.734 (0.001)
2SRI_LL_	42.509 (0.877)	0.521 (0.002)	34.750 (0.596)	0.502 (0.002)	50.881 (1.849)	23.621 (0.203)	6.586 (0.088)	0.548 (0.002)
LIML	1.426 (0.002)	1.300 (0.002)	1.788 (0.003)	1.321 (0.002)	146.244 (2.072)	7.662 (0.024)	2.481 (0.004)	12.838 (0.095)
2SLS	−3.388 (341.052)	3.900 (877.095)	−3.905 (678.671)	0.092 (180.558)	−0.758 (287.561)	−0.824 (305.084)	−1.709 (190.457)	1.202 (139.963)
2SPS_PR_	10.793 (0.016)	0.703 (0.001)	9.741 (0.012)	0.705 (0.001)	21.860 (0.032)	6.342 (0.008)	6.527 (0.013)	0.731 (0.001)
2SPS_LL_	5.765 (0.003)	0.627 (0.001)	5.326 (0.002)	0.610 (0.001)	8.445 (0.003)	4.119 (0.002)	4.345 (0.005)	0.608 (0.002)
IVW_LI_	1.213 (0.000)	1.213 (0.000)	1.213 (0.000)	1.213 (0.000)	1.213 (0.000)	1.213 (0.000)	1.213 (0.000)	1.213 (0.000)
IVW_LL_	1.374 (0.591)	1.399 (0.515)	1.292 (0.510)	1.343 (0.616)	1.297 (0.598)	1.313 (0.501)	1.701 (1.106)	1.953 (1.512)

IV, instrumental variable; IVW_LI_, inverse-variance weighted estimator with linear construction; IVW_LL_, inverse-variance weighted estimator with non-linear construction; LIML, limited information maximum likelihood; 2SLS, two-stage least square; 2SPS_PR_, two-stage predictor substitution with probit link; 2SPS_LL_, two-stage predictor substitution with log link; 2SRI_PR_, two-stage residual inclusion with probit link; 2SRI_LL_, two-stage residual inclusion with log link.

The sample size *n* is 100,000, and the simulation is iterated 200 times. The true causal risk ratio is approximately 2.96. The mean and empirical standard error (ESE) represented as “mean (ESE)” in the table are summarized. The analyses utilized 50 out of the true 500 SNPs. For the WIV methods, the datasets were split into two subsets, each containing 50,000 samples.

^a^*ρ* is a correlation coefficient between two unmeasured confounders.

**Table 4.** Summary of estimated causal risk ratio when the BMI increases from 30.5 to 37.0 without age (measured confounder) in the outcome model

Estimators	Simulation Settings							

	Using 25 Strong IVs and 25 Weak IVs							

	Bivariate Normal Distribution		Marginal Normal Distributions				Marginal t-distributions	
	
			With t-copula		With Clayton Copula	With Gumbel Copula	With Normal Copula	

	*ρ*^a^ = 0.5	*ρ*^a^ = −0.5	*ρ*^a^ = 0.5	*ρ*^a^ = −0.5	*ρ*^a^ = 0.5	*ρ*^a^ = 0.5	*ρ*^a^ = 0.5	*ρ*^a^ = −0.5
2SRI_PR_	5.077 (0.009)	2.701 (0.001)	4.486 (0.009)	2.538 (0.000)	6.956 (0.035)	4.115 (0.004)	4.224 (0.006)	2.664 (0.000)
2SRI_LL_	9.455 (0.041)	9.609 (0.011)	8.521 (0.040)	8.417 (0.008)	7.506 (0.042)	9.262 (0.030)	8.935 (0.048)	8.778 (0.003)
LIML	>999 (>999)	26.763 (55.963)	>999 (>999)	9.745 (13.836)	>999 (>999)	118.070 (356.777)	>999 (>999)	325.594 (609.598)
2SLS	−24.529 (333.940)	1.784 (21.862)	2.987 (128.939)	3.483 (14.945)	12.777 (117.642)	−7.648 (116.652)	3.674 (136.930)	3.123 (21.738)
2SPS_PR_	3.230 (0.638)	2.928 (0.756)	3.093 (0.649)	2.746 (0.752)	3.212 (0.589)	3.116 (0.685)	3.102 (0.847)	2.851 (0.873)
2SPS_LL_	2.776 (0.422)	2.602 (0.544)	2.684 (0.443)	2.466 (0.546)	2.759 (0.396)	2.696 (0.461)	2.685 (0.567)	2.524 (0.611)
IVW_LI_	1.213 (0.000)	1.213 (0.000)	1.213 (0.000)	1.213 (0.000)	1.213 (0.000)	1.213 (0.000)	1.213 (0.000)	1.213 (0.000)
IVW_LL_	1.916 (0.288)	2.342 (0.457)	1.843 (0.322)	2.180 (0.458)	1.814 (0.280)	1.927 (0.339)	2.132 (0.449)	2.321 (0.527)



Estimators	Simulation Settings							

	Using Only 50 Weak IVs							

	Bivariate Normal Distribution		Marginal Normal Distributions				Marginal t-distributions	
	
			With t-copula		With Clayton Copula	With Gumbel Copula	With Normal Copula	

	*ρ*^a^ = 0.5	*ρ*^a^ = −0.5	*ρ*^a^ = 0.5	*ρ*^a^ = −0.5	*ρ*^a^ = 0.5	*ρ*^a^ = 0.5	*ρ*^a^ = 0.5	*ρ*^a^ = −0.5

2SRI_PR_	53.288 (0.220)	1.361 (0.000)	31.470 (0.172)	1.341 (0.000)	414.946 (17.766)	18.341 (0.036)	20.675 (0.105)	1.827 (0.001)
2SRI_LL_	135.246 (2.523)	1.686 (0.003)	78.706 (2.354)	1.436 (0.003)	117.393 (4.057)	72.252 (0.598)	35.814 (0.936)	2.422 (0.005)
LIML	>999 (>999)	22.405 (20.678)	324.609 (993.139)	22.015 (20.193)	>999 (>999)	282.137 (470.460)	>999 (>999)	244.158 (489.032)
2SLS	−2.175 (2.409)	0.250 (17.268)	−2.109 (2.826)	2.206 (15.191)	−1.564 (1.472)	−3.604 (22.045)	−1.784 (4.825)	3.095 (11.335)
2SPS_PR_	15.517 (9.547)	1.683 (0.968)	13.682 (8.358)	1.798 (1.100)	24.388 (16.191)	10.536 (7.195)	17.922 (17.741)	2.970 (2.778)
2SPS_LL_	7.835 (2.892)	1.579 (0.762)	7.305 (2.606)	1.662 (0.869)	10.143 (3.684)	6.124 (2.373)	8.158 (4.568)	2.414 (1.624)
IVW_LI_	1.213 (0.000)	1.213 (0.000)	1.213 (0.000)	1.213 (0.000)	1.213 (0.000)	1.213 (0.000)	1.213 (0.000)	1.213 (0.000)
IVW_LL_	1.272 (0.464)	1.328 (0.497)	1.208 (0.443)	1.215 (0.443)	1.174 (0.475)	1.256 (0.494)	1.404 (0.738)	1.494 (0.813)

IV, instrumental variable; IVW_LI_, inverse-variance weighted estimator with linear construction; IVW_LL_, inverse-variance weighted estimator with non-linear construction; LIML, limited information maximum likelihood; 2SLS, two-stage least square; 2SPS_PR_, two-stage predictor substitution with probit link; 2SPS_LL_, two-stage predictor substitution with log link; 2SRI_PR_, two-stage residual inclusion with probit link; 2SRI_LL_, two-stage residual inclusion with log link.

The sample size *n* is 100,000, and the simulation is iterated 200 times. The true causal risk ratio is approximately 2.96. The mean and empirical standard error (ESE) represented as “mean (ESE)” in the table are summarized. The analyses utilized 50 out of the true 500 SNPs. For the WIV methods, the datasets were split into two subsets, each containing 50,000 samples.

^a^*ρ* is a correlation coefficient between two unmeasured confounders.

**Table 5.** Summary of estimated causal risk ratio when the BMI increases from 41.0 to 47.5 with age (measured confounder) included in the outcome model

Estimators	Simulation Settings							

	Using 25 Strong IVs and 25 Weak IVs							

	Bivariate Normal Distribution		Marginal Normal Distributions				Marginal t-distributions	
	
			With t-copula		With Clayton Copula	With Gumbel Copula	With Normal Copula	

	*ρ*^a^ = 0.5	*ρ*^a^ = −0.5	*ρ*^a^ = 0.5	*ρ*^a^ = −0.5	*ρ*^a^ = 0.5	*ρ*^a^ = 0.5	*ρ*^a^ = 0.5	*ρ*^a^ = −0.5
2SRI_PR_	1.989 (0.001)	1.757 (0.001)	1.958 (0.001)	1.735 (0.001)	2.067 (0.002)	1.909 (0.001)	1.877 (0.001)	1.750 (0.001)
2SRI_LL_	3.223 (0.004)	2.562 (0.004)	3.160 (0.004)	2.520 (0.004)	2.964 (0.004)	3.162 (0.003)	2.991 (0.004)	2.589 (0.004)
LIML	1.877 (0.001)	1.563 (0.001)	1.847 (0.001)	1.574 (0.001)	1.302 (0.000)	1.833 (0.001)	1.813 (0.001)	1.482 (0.000)
2SLS	1.586 (0.345)	1.534 (36.605)	1.582 (2.095)	1.676 (15.338)	1.579 (0.196)	1.577 (0.571)	1.573 (1.113)	1.489 (21.960)
2SPS_PR_	1.811 (0.001)	1.853 (0.001)	1.800 (0.001)	1.818 (0.001)	1.796 (0.001)	1.793 (0.001)	1.800 (0.001)	1.794 (0.001)
2SPS_LL_	2.671 (0.001)	2.443 (0.001)	2.588 (0.001)	2.336 (0.001)	2.662 (0.000)	2.558 (0.001)	2.598 (0.001)	2.411 (0.001)
IVW_LI_	1.159 (0.000)	1.159 (0.000)	1.159 (0.000)	1.159 (0.000)	1.159 (0.000)	1.159 (0.000)	1.159 (0.000)	1.159 (0.000)
IVW_LL_	1.993 (0.320)	2.441 (0.484)	1.927 (0.317)	2.317 (0.481)	1.890 (0.288)	1.998 (0.355)	2.225 (0.464)	2.449 (0.562)



Estimators	Simulation Settings							

	Using Only 50 Weak IVs							

	Bivariate Normal Distribution		Marginal Normal Distributions				Marginal t-distributions	
	
			With t-copula		With Clayton Copula	With Gumbel Copula	With Normal Copula	

	*ρ*^a^ = 0.5	*ρ*^a^ = −0.5	*ρ*^a^ = 0.5	*ρ*^a^ = −0.5	*ρ*^a^ = 0.5	*ρ*^a^ = 0.5	*ρ*^a^ = 0.5	*ρ*^a^ = −0.5

2SRI_PR_	1.817 (0.001)	0.861 (0.001)	1.894 (0.001)	0.971 (0.001)	1.492 (0.001)	1.936 (0.001)	1.783 (0.001)	1.052 (0.001)
2SRI_LL_	3.749 (0.002)	1.011 (0.003)	3.791 (0.003)	1.238 (0.004)	3.541 (0.002)	3.672 (0.003)	3.162 (0.002)	1.565 (0.003)
LIML	1.695 (0.001)	1.516 (0.001)	1.805 (0.001)	1.488 (0.001)	1.153 (0.000)	1.627 (0.000)	1.769 (0.001)	1.396 (0.000)
2SLS	1.767 (0.106)	17.760 (>999)	1.751 (0.107)	1.129 (110.273)	1.825 (0.070)	1.705 (0.176)	1.721 (0.882)	86.017 (>999)
2SPS_PR_	1.753 (0.000)	0.840 (0.001)	1.798 (0.001)	0.950 (0.001)	1.589 (0.000)	1.840 (0.001)	1.756 (0.001)	1.065 (0.001)
2SPS_LL_	7.375 (0.002)	0.888 (0.001)	6.718 (0.002)	1.045 (0.002)	10.603 (0.002)	5.273 (0.002)	8.163 (0.003)	1.435 (0.003)
IVW_LI_	1.159 (0.000)	1.159 (0.000)	1.159 (0.000)	1.159 (0.000)	1.159 (0.000)	1.159 (0.000)	1.159 (0.000)	1.159 (0.000)
IVW_LL_	1.374 (0.591)	1.399 (0.515)	1.292 (0.510)	1.343 (0.616)	1.297 (0.598)	1.313 (0.501)	1.701 (1.106)	1.953 (1.512)

IV, instrumental variable; IVW_LI_, inverse-variance weighted estimator with linear construction; IVW_LL_, inverse-variance weighted estimator with non-linear construction; LIML, limited information maximum likelihood; 2SLS, two-stage least square; 2SPS_PR_, two-stage predictor substitution with probit link; 2SPS_LL_, two-stage predictor substitution with log link; 2SRI_PR_, two-stage residual inclusion with probit link; 2SRI_LL_, two-stage residual inclusion with log link.

The sample size *n* is 100,000, and the simulation is iterated 200 times. The true causal risk ratio is approximately 1.85. The mean and empirical standard error (ESE) represented as “mean (ESE)” in the table are summarized. The analyses utilized 50 out of the true 500 SNPs. For the WIV methods, the datasets were split into two subsets, each containing 50,000 samples.

^a^*ρ* is a correlation coefficient between two unmeasured confounders.

**Table 6.** Summary of estimated causal risk ratio when the BMI increases from 41.0 to 47.5 without age (measured confounder) in the outcome model

Estimators	Simulation Settings							

	Using 25 Strong IVs and 25 Weak IVs							

	Bivariate Normal Distribution		Marginal Normal Distributions				Marginal t-distributions	
	
			With t-copula		With Clayton Copula	With Gumbel Copula	With Normal Copula	

	*ρ*^a^ = 0.5	*ρ*^a^ = −0.5	*ρ*^a^ = 0.5	*ρ*^a^ = −0.5	*ρ*^a^ = 0.5	*ρ*^a^ = 0.5	*ρ*^a^ = 0.5	*ρ*^a^ = −0.5
2SRI_PR_	2.086 (0.001)	1.817 (0.000)	2.052 (0.001)	1.787 (0.000)	2.253 (0.002)	1.985 (0.000)	2.040 (0.001)	1.792 (0.000)
2SRI_LL_	3.640 (0.004)	3.382 (0.002)	3.583 (0.004)	3.338 (0.002)	3.478 (0.005)	3.547 (0.002)	3.737 (0.003)	3.367 (0.001)
LIML	1.521 (0.504)	1.551 (0.386)	1.639 (1.034)	1.676 (0.368)	1.763 (0.363)	1.661 (0.472)	1.657 (0.487)	1.511 (0.483)
2SLS	1.570 (0.050)	1.542 (0.074)	1.558 (0.058)	1.521 (0.080)	1.569 (0.050)	1.559 (0.058)	1.553 (0.073)	1.527 (0.086)
2SPS_PR_	1.775 (0.058)	1.826 (0.130)	1.764 (0.073)	1.789 (0.142)	1.760 (0.057)	1.761 (0.070)	1.765 (0.097)	1.768 (0.136)
2SPS_LL_	2.776 (0.422)	2.602 (0.544)	2.684 (0.443)	2.466 (0.546)	2.759 (0.396)	2.696 (0.461)	2.685 (0.567)	2.524 (0.611)
IVW_LI_	1.159 (0.000)	1.159 (0.000)	1.159 (0.000)	1.159 (0.000)	1.159 (0.000)	1.159 (0.000)	1.159 (0.000)	1.159 (0.000)
IVW_LL_	1.916 (0.288)	2.342 (0.457)	1.843 (0.322)	2.180 (0.458)	1.814 (0.280)	1.927 (0.339)	2.132 (0.449)	2.321 (0.527)



Estimators	Simulation Settings							

	Using Only 50 Weak IVs							

	Bivariate Normal Distribution		Marginal Normal Distributions				Marginal t-distributions	
	
			With t-copula		With Clayton Copula	With Gumbel Copula	With Normal Copula	

	*ρ*^a^ = 0.5	*ρ*^a^ = −0.5	*ρ*^a^ = 0.5	*ρ*^a^ = −0.5	*ρ*^a^ = 0.5	*ρ*^a^ = 0.5	*ρ*^a^ = 0.5	*ρ*^a^ = −0.5

2SRI_PR_	1.732 (0.000)	1.436 (0.000)	1.804 (0.000)	1.487 (0.000)	1.459 (0.000)	1.851 (0.000)	1.754 (0.000)	1.667 (0.000)
2SRI_LL_	3.627 (0.001)	2.625 (0.002)	3.699 (0.001)	2.820 (0.003)	3.626 (0.001)	3.551 (0.001)	3.175 (0.001)	3.024 (0.002)
LIML	1.507 (0.668)	1.380 (0.432)	1.386 (0.668)	1.513 (0.379)	1.627 (0.477)	1.348 (0.528)	0.848 (0.684)	0.986 (0.639)
2SLS	1.791 (0.062)	1.185 (0.409)	1.780 (0.066)	1.083 (0.844)	1.830 (0.058)	1.746 (0.073)	1.774 (0.108)	1.255 (1.069)
2SPS_PR_	1.705 (0.112)	1.331 (0.387)	1.733 (0.100)	1.343 (0.466)	1.589 (0.117)	1.766 (0.087)	1.720 (0.148)	1.529 (0.420)
2SPS_LL_	7.835 (2.892)	1.579 (0.762)	7.305 (2.606)	1.662 (0.869)	10.143 (3.684)	6.124 (2.373)	8.158 (4.568)	2.414 (1.624)
IVW_LI_	1.159 (0.000)	1.159 (0.000)	1.159 (0.000)	1.159 (0.000)	1.159 (0.000)	1.159 (0.000)	1.159 (0.000)	1.159 (0.000)
IVW_LL_	1.272 (0.464)	1.328 (0.497)	1.208 (0.443)	1.215 (0.443)	1.174 (0.475)	1.256 (0.494)	1.404 (0.738)	1.494 (0.813)

IV, instrumental variable; IVW_LI_, inverse-variance weighted estimator with linear construction; IVW_LL_, inverse-variance weighted estimator with non-linear construction; LIML, limited information maximum likelihood; 2SLS, two-stage least square; 2SPS_PR_, two-stage predictor substitution with probit link; 2SPS_LL_, two-stage predictor substitution with log link; 2SRI_PR_, two-stage residual inclusion with probit link; 2SRI_LL_, two-stage residual inclusion with log link.

The sample size *n* is 100,000, and the simulation is iterated 200 times. The true causal risk ratio is approximately 1.85. The mean and empirical standard error (ESE) represented as “mean (ESE)” in the table are summarized. The analyses utilized 50 out of the true 500 SNPs. For the WIV methods, the datasets were split into two subsets, each containing 50,000 samples.

^a^*ρ* is a correlation coefficient between two unmeasured confounders.

### Overall results

When the covariate was included in the outcome model (Table 1), a clear bias was observed in the estimates of the parameters of interest obtained from the 2SLS and IVW_LI_ methods, owing to the misspecification of the outcome model. The parameter estimates of 2SRI_PR_ exhibited some bias, particularly when only weak IVs were available. In contrast, 2SPS_PR_ and IVW_LL_ yielded accurate results when strong IVs were available, but a bias arose when only weak IVs were present. Crucially, in certain cases, the parameter estimates of 2SPS_PR_ showed the opposite direction, resulting in negative estimates. This posed a significant problem, as it could lead to a misspecification of the direction of causal effects. The results of LIML showed a similar tendency to those of 2SPS_PR_ and IVW_LL_. However, all the estimates had a positive direction, even when weak IVs were employed. This represents an important distinction from the 2SPS_PR_. When the covariate was not included in the outcome model (Table 2), a noticeable bias was present in the estimates, except in 2SPS_PR_. Thus, model selection is crucial for 2SRI_PR_ and LIML, consistent with previously reported findings.^8^ Finally, regarding the results with the logit link, it should be noted that due to the misspecification of the link function, there is some bias in both the 2SPS_LL_ and 2SRI_LL_ methods under these simulation settings.

During CRR estimation when BMI increased from 30.5 to 37 (Table 3 and Table 4), both 2SRI_PR_ and 2SPS_PR_ exhibited similar patterns. However, when only weak IVs were available, a noticeable bias was observed owing to weak instrument bias. The results of IVW_LL_ and LIML also exhibited a similar pattern but with limited bias arising from weak IVs. This characteristic is well-established in the literature.^2^^,^^19^ Regarding the results obtained using the logit link, there is also some bias resulting from the misspecification of the link function. During the estimation of CRR when BMI increased from 41.0 to 47.5 (Table 5 and Table 6), all methods displayed a similar tendency. However, it is important to note that the amount of bias in all methods has decreased.

### Results focusing on 2SRI and LIML

The results of 2SRI and LIML differed significantly; the results of 2SRI_PR_ aligned more closely with those of 2SPS_PR_, whereas the results of LIML were similar to those of IVW_LL_. Thus, the methods exhibited distinct patterns, and the findings align with and justify those reported by Orihara et al.^8^^,^^21^

**Figure 2.**
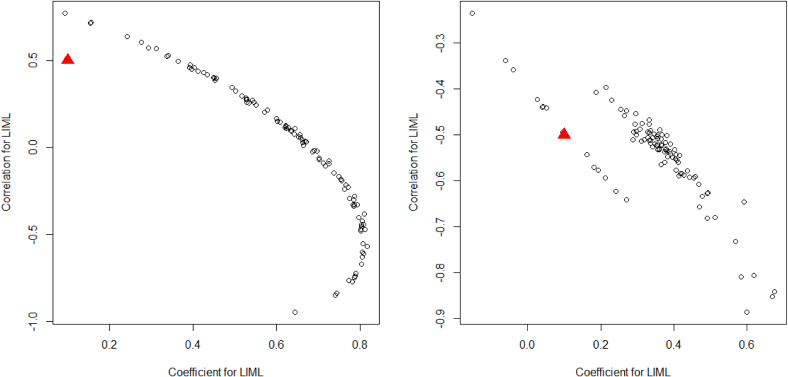
Scatter plot of the estimated treatment effect parameter and the coefficient of unmeasured confounders Left panel: Situation “Using Only 50 Weak IVs and Clayton copula including age in the outcome model”; Right panel: Situation “Using Only 50 Weak IVs and t-Distributions with normal copula (negative correlation) including age in the outcome model”; the red triangles represent the true values of each parameter. IV, instrumental variable; LIML, limited information maximum likelihood.

Several distinct patterns were observed in the simulation results. To examine these findings in greater detail, we focused on the results of LIML. Specifically, we investigated their performances in situations “Using Only 50 Weak IVs and Clayton copula including age in the outcome model” and “Using Only 50 Weak IVs and t-Distributions with normal copula (negative correlation) including age in the outcome model.” The results of the other methods also exhibited distinctive tendencies in these situations. Figure 2 presents the scatter plots of parameter estimates. Neither estimate accurately captured the true value in either situation. Thus, distinctive tendencies were observed in the estimates. For example, in the former situation, which showed strong asymmetry in unmeasured confounders, a parabolic relationship was apparent. This clearly stems from misspecification of the distribution of unmeasured confounders. However, this problem may be addressed by specifying the distribution of unmeasured confounders or employing more flexible distributions, such as copulas.^22^

## DISCUSSION

In this study, we performed simulation experiments with continuous exposure and binary outcome across various simulation settings. We conducted a comprehensive comparison of common IV methods, namely, 2SLS, 2SPS, 2SRI, LIML, IVW_LI_, and IVW_LL_. Using various simulation settings, we observed distinct trends for each method.

2SLS and IVW_LI_, which are well-known and commonly used methods, exhibited significant biases across all simulation settings. This bias was expected, because the outcome model was not specified correctly. However, we believe that this fact is not widely recognized in the fields of epidemiology, biometrics, and related disciplines. For instance, Arya et al^23^ and Karthaus et al^24^ employed the 2SLS in sensitivity analysis for the risk of unmeasured confounders, although logistic regression was used for the primary analysis. Thus, the handling of the outcome apparently differs between the primary and sensitivity analyses, given that the outcome is binary.

2SPS, which is known to introduce bias except in situations where outcome incidence is rare,^9^^,^^10^ yielded unbiased results when strong IVs were available, as observed in our simulation setting with a rare outcome. However, when only weak IVs were available, the results showed a clear bias, and in certain situations, the direction of the causal effects was opposite. This difference is crucial because it can lead to contradictory conclusions. Therefore, caution must be exercised when dealing with weak IV issues, especially when using the 2SPS. The 2SRI exhibited biased results, with an estimation tendency similar to that of the 2SPS. Theoretically, 2SRI may produce unbiased results in certain situations,^5^^,^^9^ but both the exposure and outcome models must be correctly specified for accurate detection.^8^ In this regard, the 2SPS has some advantages, because the outcome models do not necessarily need to be specified correctly. Based on these results, we found limited positive recommendations for 2SRI than for 2SPS, except for common frequency outcomes. We believe that, under proper settings, 2SPS may be a useful method.

Both LIML and IVW_LL_ tended to yield conservative results in certain situations. While they are preferable to methods that produce opposite causal effects, such as 2SRI and 2SPS, reduced statistical power may be the result. In particular, the additional simulation results highlight that LIML is sensitive to severe misspecifications of the distribution of unmeasured confounders, a weakness of this method. Furthermore, LIML requires the inclusion of as many observed covariates as possible to stabilize the results. For instance, both the mean and SD exceed 999 in some situations (Table 4). However, several steps can be undertaken to address this issue: conducting model selection^8^ and considering the distribution of unmeasured confounders. Furthermore, LIML and IVW_LL_ demonstrated minimal vulnerability to weak IV problems. This is an appealing feature of IV methods that makes them more suitable than 2SPS and 2SRI for different applications.

As only simulation experiments were conducted, several limitations of this study require consideration. First, we explored only 32 specific situations using these simulations. Although the results are extrapolatable to some extent, some uncertainties remain. For example, the results may differ when more IVs are available or when the outcome frequency deviates from the settings used. However, it is expected that the simulation settings employed in this study will be permissible within the field of MR and preference-based IV approaches. Broader and more sophisticated simulation settings are anticipated to be a key focus in future research. Second, the number of simulation iterations was limited to 200. This limitation arises from the constraints of the simulation machines, and conducting a larger number of iterations can provide more precise and expanded simulation settings. From the limited number of simulation iterations, both the results of mean and, particularly, ESE, may change as the number of iterations increases. These limitations require consideration when interpreting the findings of this study, and further research using larger and more diverse simulation settings is warranted.

In future work, considering that all IVs applied in IV methods are assumed valid, it is necessary to conduct simulation experiments under conditions that violate IV assumptions. Specifically, addressing violations related to ‘IVs being associated with the outcome only through the exposure,’ ‘IVs being independent of unmeasured confounders,’ and ‘the homogeneity assumption’ is crucial. Such assumption violations are discussed widely in many IV contexts.^1^^,^^2^ Conducting simulation experiments in settings where these violations are either slight or severe represents an interesting direction for future work, building upon this manuscript. Additionally, whereas the present manuscript assumes only time-fixed exposure situations, time-varying exposures are commonly encountered, especially in MR.^25^^,^^26^ Additional simulation experiments under time-varying exposure situations need to be implemented in future work.

### Conclusion

In summary, the findings indicate that no panacea is available for the bias associated with IV methods; however, some strategies can be recommended. First, we recommend the use of multiple IV methods. For example, when planning clinical research, LIML is employed for primary analyses, whereas 2SPS is used for sensitivity analyses, or vice versa. Second, if strong IVs are available, 2SPS might be preferred; however, with only weak IVs, LIML or IVW_LL_ may be better choices. Third, particularly for 2SRI and LIML, it is recommended to adjust for some observed confounders by including them in the models. However, because there is a risk that the confounders may introduce collider bias,^2^ a very careful selection process is necessary. Most importantly, as IV methods require adherence to four conditions outlined at the start of this paper, these conditions must be carefully verified.

## SUPPLEMENTARY MATERIAL

eMaterial 1. Brief introduction of instrumental variable methods

eMaterial 2. Simulation results for causal risk difference and detail the differences from causal risk ratio

eTable 1. Summary of estimated causal risk ratio when the BMI increases from 18.5 to 25.0 with age (measured confounder) included in the outcome model

eTable 2. Summary of estimated causal risk ratio when the BMI increases from 18.5 to 25.0 without age (measured confounder) in the outcome model

eTable 3. Summary of estimated causal risk ratio when the BMI increases from 53.0 to 59.5 with age (measured confounder) included in the outcome model

eTable 4. Summary of estimated causal risk ratio when the BMI increases from 53.0 to 59.5 without age (measured confounder) in the outcome model

eTable 5. Summary of estimated causal risk differences when the BMI increases from 18.5 to 25.0 with age (measured confounder) included in the outcome model

eTable 6. Summary of estimated causal risk differences when the BMI increases from 18.5 to 25.0 without age (measured confounder) in the outcome model

eTable 7. Summary of estimated causal risk differences when the BMI increases from 30.5 to 37.0 with age (measured confounder) included in the outcome model

eTable 8. Summary of estimated causal risk differences when the BMI increases from 30.5 to 37.0 without age (measured confounder) in the outcome model

eTable 9. Summary of estimated causal risk differences when the BMI increases from 41.0 to 47.5 with age (measured confounder) included in the outcome model

eTable 10. Summary of estimated causal risk differences when the BMI increases from 41.0 to 47.5 without age (measured confounder) in the outcome model

eTable 11. Summary of estimated causal risk differences when the BMI increases from 53.0 to 59.5 with age (measured confounder) included in the outcome model

eTable 12. Summary of estimated causal risk differences when the BMI increases from 53.0 to 59.5 without age (measured confounder) in the outcome model
