# Supplementary material for: Comparison of Instrumental Variable Methods With Continuous Exposure and Binary Outcome: A Simulation Study
Source: J Epidemiol. 2025 Jan 5;35(1):11–20. doi: 10.2188/jea.JE20230271 (PMC11637812; doi:10.2188/jea.JE20230271)
Supplement: Supplementary file 1 [file je-35-011-s001.pdf]

eMaterial 1. Brief introduction of instrumental variable methods

Let n be the sample size and $i = 1, 2, \ldots, n$ denote subjects of an *i.i.d.* sample. Here, $\boldsymbol{Z}_i = (Z_{i1}, \ldots, Z_{iK})^\top$, $\boldsymbol{X}_i$, T_i, Y_i, and $(V_i, U_i)^\top$ represent instrumental variables (IVs), measured confounders, an exposure variable, an outcome, and unmeasured confounders, respectively.

Two-stage least square / two-stage predictor substitution

In the first stage, a regression model is prepared, regressing the IVs (and the measured confounders) on the exposure variable:

$$\mathrm{E}[T|\boldsymbol{z}_i, \boldsymbol{x}_i] = \alpha_0 + \boldsymbol{z}_i^\top \alpha_z + \boldsymbol{x}_i^\top \alpha_x = \boldsymbol{w}_i^\top \boldsymbol{\alpha}$$

Then, estimate $\boldsymbol{\alpha}$ using methods such as the ordinary least squares (OLS) estimator:

$$\widehat{\boldsymbol{\alpha}} = \left(\sum_{i=1}^{n} \boldsymbol{w}_i \boldsymbol{w}_i^\top\right)^{-1} \sum_{i=1}^{n} \boldsymbol{w}_i t_i,$$

and obtaining predictors $\hat{T}_i = \boldsymbol{w}_i^\top \widehat{\boldsymbol{\alpha}}$.

In the second stage, a regression model regressing the predictors (and the measured confounders) on the outcome variable is prepared:

$$g(E[Y|\hat{t}_i, \boldsymbol{x}_i])^{-1} = \beta_0 + \hat{t}_i \beta_t + \boldsymbol{x}_i^\top \beta_x,$$

where g is some link function. For two-stage least square (2SLS), the identity link and the OLS is commonly used; this is origin of the name “2SLS.” Note that for 2SLS, model specification regarding the first step is not important to derive the consistent estimator for β.

For implementing 2SLS, R function ivreg::ivreg[1] is commonly used. For two-stage predictor

substitution (2SPS), recently, R function OneSampleMR::tsps[2] has been developed. The function has the same syntax as ivreg::ivreg and are easy to implement. These functions can derive not only point estimates but also confidence intervals. However, OneSampleMR::tsps only supports the logistic link ($g^{-1} = logit$; logistic regression model) and log link ($g^{-1} = log$; log-linear model), and thus cannot implement probit model.

Two-stage residual inclusion

The first stage process is similar to 2SLS and 2SPS; the difference lies in deriving residuals for the exposure variable: $\widehat{U}_i = T_i - \boldsymbol{w}_i^\top \widehat{\boldsymbol{\alpha}}$.

In the second stage, a regression model is prepared, regressing the exposure variable and the residuals (and the measured confounders) on the outcome variable:

$$g(E[Y|t_i, \hat{u}_i, \boldsymbol{x}_i])^{-1} = \beta_0 + t_i\beta_t + \hat{u}_i\beta_u + \boldsymbol{x}_i^\top \beta_x,$$

where g is some link function.

For two-stage residual inclusion (2SRI), R function OneSampleMR::tsri[2] also can be applied. The function can derive not only point estimates but also confidence intervals. However, OneSampleMR::tsri only supports the logistic link ($g^{-1} = logit$; logistic regression model) and log link ($g^{-1} = log$; log-linear model), and thus cannot implement probit model.

Limited information maximum likelihood

As mentioned in the main manuscript, the objective of limited information maximum likelihood (LIML) is similar to that of 2SPS and 2SRI. However, its estimation process differs from these methods. We assume that the unmeasured confounders follow a bivariate distribution; commonly, a bivariate normal distribution is assumed:

$$\begin{pmatrix} V_i \\ U_i \end{pmatrix} \sim^{i.i.d.} N\left(\mathbf{0}_2, \begin{pmatrix} \sigma_v^2 & \rho\sigma_v \\ \sigma_{uv} & 1 \end{pmatrix}\right).$$

Additionally, assume that the exposure and outcome models can be expressed as follows:

$$T_i = \boldsymbol{w}_i^\top \boldsymbol{\alpha} + V_i\,, \quad Y_i = \mathbf{1}\{U_i \geq \beta_0 + t_i\beta_t + \boldsymbol{x}_i^\top \beta_x\},$$

where $\mathbf{1}\{A\}$ denotes the indicator function, which equals 1 when A is true, and 0 otherwise. Note that since U_i follows the standard normal distribution, Y_i follows the probit model.

Under these assumptions, the likelihood for the exposure and outcome (conditional on the IVs and measured confounders) can be expressed as follows:

$$\begin{aligned} f(t_i, y_i | \boldsymbol{z}_i, \boldsymbol{x}_i) &= f(y_i | t_i, \boldsymbol{z}_i, \boldsymbol{x}_i; \boldsymbol{\beta}, \sigma_v^2, \rho) f(t_i | \boldsymbol{z}_i, \boldsymbol{x}_i; \boldsymbol{\alpha}) \\ &= \Phi\left(\frac{\beta_0 + t_i\beta_t + \boldsymbol{x}_i^\top \beta_x + \rho(t_i - \boldsymbol{w}_i^\top \boldsymbol{\alpha})}{\sqrt{(1-\rho)^2}}\right)^{y_i} \\ &\quad \times \left(1 - \Phi\left(\frac{\beta_0 + t_i\beta_t + \boldsymbol{x}_i^\top \beta_x + \rho(t_i - \boldsymbol{w}_i^\top \boldsymbol{\alpha})}{\sqrt{(1-\rho)^2}}\right)\right)^{1-y_i} \phi(t_i - \boldsymbol{w}_i^\top \boldsymbol{\alpha}), \end{aligned}$$

where Φ and ϕ are the cumulative distribution and density functions of the standard normal distribution, respectively. Specifically, the above model is called the “Rivers-Vuong model.”[3] To estimate the parameters, one maximizes the ‘joint’ likelihood to obtain the maximum likelihood estimators $\widehat{\boldsymbol{\alpha}}$ and $\widehat{\boldsymbol{\beta}}$. More details appear in Section 2 of Orihara et al.[4]

Unfortunately, to the best of our knowledge, there are no R functions available to implement LIML for dichotomous outcomes. While the ivmodel::LIML function does exist, it can only be applied to situations with continuous exposure and outcomes.

Inverse-variance weighted estimator

For the inverse-variance weighted (IVW) estimator, two datasets are required. In the simulation experiments in the main manuscript, one dataset is split into two datasets. A regression model is prepared using one of these datasets, regressing each IV on the exposure variable:

$$\mathrm{E}[T|z_{ik}] = \alpha_{0k} + z_{ik}\alpha_{zk} + \boldsymbol{x}_i^\top \alpha_{xk} = \boldsymbol{w}_{ik}^\top \boldsymbol{\alpha}_k$$

Then, $\boldsymbol{\alpha_k}$ is estimated using methods like the OLS estimator:

$$\widehat{\boldsymbol{\alpha}}_{\boldsymbol{k}} = \left(\sum_{\boldsymbol{i=1}}^{\boldsymbol{n}} \boldsymbol{w}_{\boldsymbol{ik}} \boldsymbol{w}_{ik}^\top\right)^{-\boldsymbol{1}} \sum_{\boldsymbol{i=1}}^{\boldsymbol{n}} \boldsymbol{w}_{\boldsymbol{ik}} t_i.$$

Using the second dataset, another regression model is prepared, regressing each IV on the outcome variable:

$$g(E[Y|z_{ik}])^{-1} = \beta_{0k} + z_{ik}\beta_{zk} + \boldsymbol{x}_i^\top \boldsymbol{\beta}_{xk},$$

where g is some link function.

For a linear model (IVW$_{\mathrm{LI}}$; where g is the identity link), the IVW estimator $\hat{\theta}_{IVW_{LI}}$ is given by

$$\hat{\theta}_{IVW_{LI}} = \frac{\sum_{k=1}^{K} \frac{\hat{\beta}_{zk}\hat{\alpha}_{zk}}{se(\hat{\beta}_{zk})^2}}{\sum_{k=1}^{K} \frac{\hat{\alpha}_{zk}^{\,2}}{se(\hat{\beta}_{zk})^2}},$$

where $se(\hat{\beta}_{zk})$ is the standard error of each $\hat{\beta}_{zk}$. Since both the numerator and denominator are

divided by the variance of $\hat{\beta}_{zk}$, this is origin of the name "IVW." Other link functions, like logit and log, can also be implemented similarly.

IVW methods can be implemented using the lm and glm functions in R. Additionally, for two-sample MR situations, the TwoSampleMR library offers many analysis functions.

Additional information

Some of the IV methods discussed in the main manuscript cannot be implemented using standard R functions. The following GitHub repository provides scripts to implement 2SRI with the probit link ($g^{-1} = \Phi^{-1}$) and LIML as explained above:

- https://github.com/SOrihara/Instrumental-Variable-Estimation-of-Causal-Effects-with-Applying-Some-Model-Selection-Procedures

Note that the functions in the provided link can only derive point estimates. The functions will be updated in the future.

REFERENCES

1. Fox J, Kleiber C, Zeileis A. *ivreg: Instrumental-Variables Regression by '2SLS', '2SM', or '2SMM', with Diagnostics*; 2023. https://CRAN.R-project.org/package=ivreg.
2. Palmer T, Spiller W, Sanderson E. *OneSampleMR: One Sample Mendelian Randomization and Instrumental Variable Analyses*; 2023. https://CRAN.R-project.org/package=OneSampleMR.
3. Rivers D, Vuong QH. Limited information estimators and exogeneity tests for simultaneous probit models. *Journal of Econometrics.* 1988;39:3, 347-366.
4. Orihara S, Goto A, Taguri M. Instrumental variable estimation of causal effects with applying some model selection procedures under binary outcomes. *Behaviormetrika*. 2023;50:1, 241-262.

eMaterial 2. Simulation results for causal risk difference and detail the differences from causal risk ratio

Detailed differences between causal risk ratio and causal risk difference

As Hernán and Robins (2020) mention:

> *Each effect measure may be used for different purposes. For example, imagine a large population in which 3 in a million individuals would develop the outcome if treated, and 1 in a million individuals would develop the outcome if untreated. The causal risk ratio is 3, and the causal risk difference is 0.000002. The causal risk ratio (multiplicative scale) is used to compute how many times treatment, relative to no treatment, increases the disease risk. The causal risk difference (additive scale) is used to compute the absolute number of cases of the disease attributable to the treatment. (Hernán and Robins, 2020; p.8)*

Thus, causal risk difference (CRD) is preferable when interested in the absolute number of affected patients. However, "The use of either the multiplicative or additive scale will depend on the goal of the inference." (Hernán and Robins, 2020; p.8)

Regarding some estimating methods described in the main manuscript, their parameter estimates can be interpreted as either the causal risk ratio (CRR) or CRD.

For CRR, the parameter estimates of IVW_{LL}, and $2SPS_{LL}/2SRI_{LL}$ without measured confounders can be interpreted on a log-scale risk ratio. Actually,

$$\log\left(\frac{\hat{p}(t_2)}{\hat{p}(t_1)}\right) = \log(\hat{p}(t_2)) - \log(\hat{p}(t_1)) = \log(exp(\hat{\alpha}_0 + t_2\hat{\alpha}_t)) - \log(exp(\hat{\alpha}_0 + t_1\hat{\alpha}_t))$$

$$= \hat{\alpha}_0 + t_2\hat{\alpha}_t - (\hat{\alpha}_0 + t_1\hat{\alpha}_t) = (t_2 - t_1)\hat{\alpha}_t.$$

This is the reason why the log-linear model is preferred for analyzing CRR. The other methods described in the main manuscript do not offer this advantageous feature, particularly $2SPS_{LL}$/$2SRI_{LL}$ with measured confounders, due to the application of G-formula based methods in our simulations.

For CRD, the parameter estimates of $2SLS/IVW_{LI}$ can be interpreted as risk difference. Actually,

$$\hat{p}(t_2) - \hat{p}(t_1) = \hat{\alpha}_0 + t_2\hat{\alpha}_t - (\hat{\alpha}_0 + t_1\hat{\alpha}_t) = (t_2 - t_1)\hat{\alpha}_t.$$

This is the reason why the linear model is preferrable for analyzing CRD. The other methods described in the main manuscript do not offer this advantageous feature. Note that for 2SLS, the G-formula based method (ie, with measured confounders) also demonstrates this feature due to the additive nature of "$\mathbf{\Sigma}_{i=1}^{n}$".

The estimated parameters of interest (ie, the exposure variables) are summarized in eTable 1, eTable 2, eTable 3, eTable 4, eTable 5, eTable 6, eTable 7, eTable 8, eTable 9, eTable 10, eTable 11, and eTable 12.

eTable 1. Summary of estimated causal risk ratio when the BMI increases from 18.5 to 25.0 with age (measured confounder) included in the outcome model

Estimators	Simulation Settings							
	Using 25 Strong IVs and 25 Weak IVs							
	Bivariate Normal Distribution		Marginal Normal Distributions				Marginal t-distributions	
			With t-copula		With Clayton Copula	With Gumbel Copula	With Normal Copula	
	$\rho^a = 0.5$	$\rho^a = -0.5$	$\rho^a = 0.5$	$\rho^a = -0.5$	$\rho^a = 0.5$	$\rho^a = 0.5$	$\rho^a = 0.5$	$\rho^a = -0.5$
$2SRI_{PR}$	6.211(0.064)	2.959(0.009)	4.990(0.039)	2.797(0.007)	7.671(0.229)	4.769(0.027)	3.793(0.023)	2.723(0.011)
$2SRI_{LL}$	7.029(0.188)	4.591(0.066)	6.328(0.125)	4.376(0.052)	6.570(0.170)	6.861(0.123)	4.375(0.087)	3.577(0.054)
LIML	0.996(0.000)	0.998(0.001)	1.040(0.000)	1.106(0.001)	3.671(0.013)	3.311(0.009)	3.080(0.007)	8.613(0.108)
2SLS	0.774(161.431)	-0.996(603.268)	0.202(15.774)	0.877(162.918)	0.313(18.851)	0.397(28.618)	-0.555(188.359)	0.140(34.532)

$2SPS_{PR}$	4.961(0.010)	3.075(0.008)	4.264(0.008)	2.904(0.006)	5.030(0.009)	4.266(0.009)	3.587(0.008)	2.755(0.007)
$2SPS_{LL}$	2.440(0.001)	2.099(0.001)	2.339(0.001)	2.034(0.001)	2.456(0.001)	2.318(0.001)	2.220(0.001)	2.003(0.001)
IVW_{LI}	1.351 (0.000)	1.351 (0.000)	1.351 (0.000)	1.351 (0.000)	1.351 (0.000)	1.351 (0.000)	1.351 (0.000)	1.351 (0.000)
IVW_{LL}	1.993 (0.320)	2.441 (0.484)	1.927 (0.317)	2.317 (0.481)	1.890 (0.288)	1.998 (0.355)	2.225 (0.464)	2.449 (0.562)

IV, instrumental variable; IVW_{LI}, inverse-variance weighted estimator with linear construction; IVW_{LL}, inverse-variance weighted estimator with non-linear construction; LIML, limited information maximum likelihood; 2SLS, two-stage least square; $2SPS_{PR}$, two-stage predictor substitution with probit link; $2SPS_{LL}$, two-stage predictor substitution with log link; $2SRI_{PR}$, two-stage residual inclusion with probit link; $2SRI_{LL}$, two-stage residual inclusion with log link.

The sample size n is 100,000, and the simulation is iterated 200 times. The true causal risk ratio is approximately 5.44. The mean and empirical standard error (ESE) represented as "mean (ESE)" in the table are summarized. The analyses utilized 50 out of the true 500 SNPs. For the WIV methods, the datasets were split into two subsets, each containing 50,000 samples.

[a] ρ is a correlation coefficient between two unmeasured confounders.

eTable 1 (continued). Summary of estimated causal risk ratio when the BMI increases from 18.5 to 25.0 with age (measured confounder) included in the outcome model

Estimators	Simulation Settings							
	Using Only 50 Weak IVs							
	Bivariate Normal Distribution		Marginal Normal Distributions				Marginal t-distributions	
			With t-copula		With Clayton Copula	With Gumbel Copula	With Normal Copula	
	$\rho^a = 0.5$	$\rho^a = -0.5$	$\rho^a = 0.5$	$\rho^a = -0.5$	$\rho^a = 0.5$	$\rho^a = 0.5$	$\rho^a = 0.5$	$\rho^a = -0.5$
$2SRI_{PR}$	7.847(0.360)	0.762(0.001)	12.836(0.323)	0.756(0.001)	32.732(10.575)	12.098(0.197)	2.017(0.015)	0.781(0.001)
$2SRI_{LL}$	4.643(0.302)	0.605(0.001)	7.113(0.607)	0.588(0.001)	9.038(0.950)	14.535(0.490)	0.694(0.004)	0.618(0.001)
LIML	0.842(0.001)	0.893(0.001)	0.832(0.001)	0.899(0.001)	5.599(0.088)	1.402(0.004)	0.974(0.001)	0.849(0.003)
2SLS	0.512(4.471)	0.681(43.037)	0.515(0.271)	0.850(23.629)	0.551(0.110)	0.472(1.505)	1.056(161.435)	0.825(15.218)

$2SPS_{PR}$	7.094(0.091)	0.763(0.000)	14.667(0.098)	0.754(0.001)	7.065(0.097)	8.524(0.047)	1.870(0.006)	0.783(0.000)
$2SPS_{LL}$	4.060(0.006)	0.379(0.003)	4.287(0.002)	0.387(0.001)	5.769(0.009)	3.316(0.002)	2.040(0.005)	0.302(0.001)
IVW_{LI}	1.351 (0.000)	1.351 (0.000)	1.351 (0.000)	1.351 (0.000)	1.351 (0.000)	1.351 (0.000)	1.351 (0.000)	1.351 (0.000)
IVW_{LL}	1.374 (0.591)	1.399 (0.515)	1.292 (0.510)	1.343 (0.616)	1.297 (0.598)	1.313 (0.501)	1.701 (1.106)	1.953 (1.512)

IV, instrumental variable; IVW_{LI}, inverse-variance weighted estimator with linear construction; IVW_{LL}, inverse-variance weighted estimator with non-linear construction; LIML, limited information maximum likelihood; 2SLS, two-stage least square; $2SPS_{PR}$, two-stage predictor substitution with probit link; $2SPS_{LL}$, two-stage predictor substitution with log link; $2SRI_{PR}$, two-stage residual inclusion with probit link; $2SRI_{LL}$, two-stage residual inclusion with log link.

The sample size n is 100,000, and the simulation is iterated 200 times. The true causal risk ratio is approximately 5.44. The mean and empirical standard error (ESE) represented as “mean (ESE)” in the table are summarized. The analyses utilized 50 out of the true 500 SNPs. For the WIV methods, the datasets were split into two subsets, each containing 50,000 samples.

[a] ρ is a correlation coefficient between two unmeasured confounders.

eTable 2. Summary of estimated causal risk ratio when the BMI increases from 18.5 to 25.0 without age (measured confounder) in the outcome model

**Estimators**	**Simulation Settings**							
	**Using 25 Strong IVs and 25 Weak IVs**							
	**Bivariate Normal Distribution**		**Marginal Normal Distributions**				**Marginal t-distributions**	
			**With t-copula**		**With Clayton Copula**	**With Gumbel Copula**	**With Normal Copula**	
	$\boldsymbol{\rho}^{a} = \mathbf{0.5}$	$\boldsymbol{\rho}^{a} = -\mathbf{0.5}$	$\boldsymbol{\rho}^{a} = \mathbf{0.5}$	$\boldsymbol{\rho}^{a} = -\mathbf{0.5}$	$\boldsymbol{\rho}^{a} = \mathbf{0.5}$	$\boldsymbol{\rho}^{a} = \mathbf{0.5}$	$\boldsymbol{\rho}^{a} = \mathbf{0.5}$	$\boldsymbol{\rho}^{a} = -\mathbf{0.5}$
$2SRI_{PR}$	9.085(0.085)	3.520(0.002)	6.970(0.050)	3.137(0.001)	12.865(0.547)	6.510(0.031)	5.512(0.039)	3.182(0.000)
$2SRI_{LL}$	10.936(0.358)	10.986(0.031)	9.746(0.200)	8.631(0.017)	9.886(0.326)	11.143(0.184)	6.938(0.242)	5.913(0.008)
LIML	> 999(> 999)	> 999(> 999)	> 999(> 999)	> 999(> 999)	> 999(> 999)	> 999(> 999)	> 999(> 999)	> 999(> 999)
2SLS	0.403(0.068)	0.330(0.214)	0.381(0.099)	0.665(3.823)	0.400(0.073)	0.384(0.092)	0.333(0.301)	0.363(0.369)

$2SPS_{PR}$	7.623(3.798)	5.743(3.184)	6.915(3.257)	5.099(3.124)	7.559(3.101)	7.126(3.716)	7.242(5.154)	5.871(4.364)
$2SPS_{LL}$	2.776(0.422)	2.602(0.544)	2.684(0.443)	2.466(0.546)	2.759(0.396)	2.696(0.461)	2.685(0.567)	2.524(0.611)
IVW_{LI}	1.351 (0.000)	1.351 (0.000)	1.351 (0.000)	1.351 (0.000)	1.351 (0.000)	1.351 (0.000)	1.351 (0.000)	1.351 (0.000)
IVW_{LL}	1.916 (0.288)	2.342 (0.457)	1.843 (0.322)	2.180 (0.458)	1.814 (0.280)	1.927 (0.339)	2.132 (0.449)	2.321 (0.527)

IV, instrumental variable; IVW_{LI}, inverse-variance weighted estimator with linear construction; IVW_{LL}, inverse-variance weighted estimator with non-linear construction; LIML, limited information maximum likelihood; 2SLS, two-stage least square; $2SPS_{PR}$, two-stage predictor substitution with probit link; $2SPS_{LL}$, two-stage predictor substitution with log link; $2SRI_{PR}$, two-stage residual inclusion with probit link; $2SRI_{LL}$, two-stage residual inclusion with log link.

The sample size n is 100,000, and the simulation is iterated 200 times. The true causal risk ratio is approximately 5.44. The mean and empirical standard error (ESE) represented as "mean (ESE)" in the table are summarized. The analyses utilized 50 out of the true 500 SNPs. For the WIV methods, the datasets were split into two subsets, each containing 50,000 samples.

[a] ρ is a correlation coefficient between two unmeasured confounders.

eTable 2 (continued). Summary of estimated causal risk ratio when the BMI increases from 18.5 to 25.0 without age (measured confounder) in the outcome model

**Estimators**	**Simulation Settings**							
	**Using Only 50 Weak IVs**							
	**Bivariate Normal Distribution**		**Marginal Normal Distributions**				**Marginal t-distributions**	
			**With t-copula**		**With Clayton Copula**	**With Gumbel Copula**	**With Normal Copula**	
	$\rho^a = 0.5$	$\rho^a = -0.5$	$\rho^a = 0.5$	$\rho^a = -0.5$	$\rho^a = 0.5$	$\rho^a = 0.5$	$\rho^a = 0.5$	$\rho^a = -0.5$
$2SRI_{PR}$	52.027(3.501)	1.053(0.000)	4.039(0.112)	0.944(0.000)	132.471(43.499)	16.036(0.432)	1.428(0.011)	1.048(0.000)
$2SRI_{LL}$	19.931(1.759)	0.653(0.000)	3.236(0.117)	0.575(0.001)	19.842(2.534)	35.505(1.329)	0.684(0.004)	0.607(0.001)
LIML	> 999(> 999)	> 999(> 999)	> 999(> 999)	> 999(> 999)	> 999(> 999)	> 999(> 999)	> 999(> 999)	> 999(> 999)
2SLS	0.542(0.024)	> 999(> 999)	0.536(0.047)	-0.653(8.195)	0.554(0.025)	0.522(0.044)	0.554(0.227)	1.335(7.782)

2SPS$_{PR}$	> 999(> 999)	2.641(3.552)	> 999(> 999)	3.074(4.240)	> 999(> 999)	> 999(> 999)	> 999(> 999)	18.523(69.063)
2SPS$_{LL}$	7.835(2.892)	1.579(0.762)	7.305(2.606)	1.662(0.869)	10.143(3.684)	6.124(2.373)	8.158(4.568)	2.414(1.624)
IVW$_{LI}$	1.351 (0.000)	1.351 (0.000)	1.351 (0.000)	1.351 (0.000)	1.351 (0.000)	1.351 (0.000)	1.351 (0.000)	1.351 (0.000)
IVW$_{LL}$	1.272 (0.464)	1.328 (0.497)	1.208 (0.443)	1.215 (0.443)	1.174 (0.475)	1.256 (0.494)	1.404 (0.738)	1.494 (0.813)

IV, instrumental variable; IVW$_{LI}$, inverse-variance weighted estimator with linear construction; IVW$_{LL}$, inverse-variance weighted estimator with non-linear construction; LIML, limited information maximum likelihood; 2SLS, two-stage least square; 2SPS$_{PR}$, two-stage predictor substitution with probit link; 2SPS$_{LL}$, two-stage predictor substitution with log link; 2SRI$_{PR}$, two-stage residual inclusion with probit link; 2SRI$_{LL}$, two-stage residual inclusion with log link.

The sample size n is 100,000, and the simulation is iterated 200 times. The true causal risk ratio is approximately 5.44. The mean and empirical standard error (ESE) represented as “mean (ESE)” in the table are summarized. The analyses utilized 50 out of the true 500 SNPs. For the WIV methods, the datasets were split into two subsets, each containing 50,000 samples.

[a] ρ is a correlation coefficient between two unmeasured confounders.

eTable 3. Summary of estimated causal risk ratio when the BMI increases from 53.0 to 59.5 with age (measured confounder) included in the outcome model

Estimators	Simulation Settings							
	Using 25 Strong IVs and 25 Weak IVs							
	Bivariate Normal Distribution		Marginal Normal Distributions				Marginal t-distributions	
			With t-copula		With Clayton Copula	With Gumbel Copula	With Normal Copula	
	$\boldsymbol{\rho}^{\mathrm{a}} = \mathbf{0.5}$	$\boldsymbol{\rho}^{\mathrm{a}} = -\mathbf{0.5}$	$\boldsymbol{\rho}^{\mathrm{a}} = \mathbf{0.5}$	$\boldsymbol{\rho}^{\mathrm{a}} = -\mathbf{0.5}$	$\boldsymbol{\rho}^{\mathrm{a}} = \mathbf{0.5}$	$\boldsymbol{\rho}^{\mathrm{a}} = \mathbf{0.5}$	$\boldsymbol{\rho}^{\mathrm{a}} = \mathbf{0.5}$	$\boldsymbol{\rho}^{\mathrm{a}} = -\mathbf{0.5}$
$2SRI_{PR}$	1.160(0.000)	1.276(0.000)	1.178(0.000)	1.284(0.000)	1.137(0.000)	1.183(0.000)	1.192(0.000)	1.250(0.000)
$2SRI_{LL}$	1.421(0.001)	1.484(0.001)	1.445(0.001)	1.500(0.001)	1.493(0.001)	1.411(0.001)	1.402(0.001)	1.453(0.001)
LIML	1.101(0.000)	1.190(0.000)	1.133(0.000)	1.201(0.000)	1.103(0.000)	1.217(0.000)	1.217(0.000)	1.002(0.000)
2SLS	1.276(0.035)	1.271(0.242)	1.273(0.038)	1.273(1.178)	1.276(0.032)	1.273(0.040)	1.272(0.044)	1.263(0.958)

$2SPS_{PR}$	1.175(0.000)	1.281(0.000)	1.188(0.000)	1.291(0.000)	1.164(0.000)	1.189(0.000)	1.197(0.000)	1.253(0.000)
$2SPS_{LL}$	2.803(0.001)	2.652(0.001)	2.710(0.000)	2.533(0.001)	2.762(0.000)	2.692(0.001)	2.813(0.001)	2.669(0.001)
IVW_{LI}	1.123 (0.000)	1.123 (0.000)	1.123 (0.000)	1.123 (0.000)	1.123 (0.000)	1.123 (0.000)	1.123 (0.000)	1.123 (0.000)
IVW_{LL}	1.993 (0.320)	2.441 (0.484)	1.927 (0.317)	2.317 (0.481)	1.890 (0.288)	1.998 (0.355)	2.225 (0.464)	2.449 (0.562)

IV, instrumental variable; IVW_{LI}, inverse-variance weighted estimator with linear construction; IVW_{LL}, inverse-variance weighted estimator with non-linear construction; LIML, limited information maximum likelihood; 2SLS, two-stage least square; $2SPS_{PR}$, two-stage predictor substitution with probit link; $2SPS_{LL}$, two-stage predictor substitution with log link; $2SRI_{PR}$, two-stage residual inclusion with probit link; $2SRI_{LL}$, two-stage residual inclusion with log link.

The sample size n is 100,000, and the simulation is iterated 200 times. The true causal risk ratio is approximately 1.25. The mean and empirical standard error (ESE) represented as “mean (ESE)” in the table are summarized. The analyses utilized 50 out of the true 500 SNPs. For the WIV methods, the datasets were split into two subsets, each containing 50,000 samples.

[a] ρ is a correlation coefficient between two unmeasured confounders.

eTable 3 (continued). Summary of estimated causal risk ratio when the BMI increases from 53.0 to 59.5 with age (measured confounder) included in the outcome model

Estimators	Simulation Settings							
	Using Only 50 Weak IVs							
	Bivariate Normal Distribution		Marginal Normal Distributions				Marginal t-distributions	
			With t-copula		With Clayton Copula	With Gumbel Copula	With Normal Copula	
	$\rho^a = 0.5$	$\rho^a = -0.5$	$\rho^a = 0.5$	$\rho^a = -0.5$	$\rho^a = 0.5$	$\rho^a = 0.5$	$\rho^a = 0.5$	$\rho^a = -0.5$
$2SRI_{PR}$	1.011(0.000)	1.025(0.001)	1.023(0.000)	1.111(0.001)	1.001(0.000)	1.043(0.000)	1.041(0.000)	1.100(0.001)
$2SRI_{LL}$	1.046(0.000)	1.297(0.002)	1.063(0.000)	1.337(0.003)	1.030(0.000)	1.109(0.000)	1.086(0.000)	1.242(0.001)
LIML	1.065(0.000)	1.127(0.000)	1.050(0.000)	1.110(0.000)	1.003(0.000)	1.046(0.000)	1.094(0.000)	1.010(0.000)
2SLS	1.316(0.020)	3.809(867.081)	1.313(0.020)	1.357(46.485)	1.326(0.013)	1.303(0.028)	1.302(0.059)	1.373(42.087)

$2SPS_{PR}$	1.022(0.000)	1.007(0.001)	1.027(0.000)	1.103(0.001)	1.005(0.000)	1.058(0.000)	1.046(0.000)	1.100(0.000)
$2SPS_{LL}$	9.197(0.001)	1.355(0.003)	9.111(0.001)	1.869(0.004)	13.633(0.005)	7.263(0.002)	13.844(0.003)	3.610(0.007)
IVW_{LI}	1.123 (0.000)	1.123 (0.000)	1.123 (0.000)	1.123 (0.000)	1.123 (0.000)	1.123 (0.000)	1.123 (0.000)	1.123 (0.000)
IVW_{LL}	1.374 (0.591)	1.399 (0.515)	1.292 (0.510)	1.343 (0.616)	1.297 (0.598)	1.313 (0.501)	1.701 (1.106)	1.953 (1.512)

IV, instrumental variable; IVW_{LI}, inverse-variance weighted estimator with linear construction; IVW_{LL}, inverse-variance weighted estimator with non-linear construction; LIML, limited information maximum likelihood; 2SLS, two-stage least square; $2SPS_{PR}$, two-stage predictor substitution with probit link; $2SPS_{LL}$, two-stage predictor substitution with log link; $2SRI_{PR}$, two-stage residual inclusion with probit link; $2SRI_{LL}$, two-stage residual inclusion with log link.

The sample size n is 100,000, and the simulation is iterated 200 times. The true causal risk ratio is approximately 1.25. The mean and empirical standard error (ESE) represented as "mean (ESE)" in the table are summarized. The analyses utilized 50 out of the true 500 SNPs. For the WIV methods, the datasets were split into two subsets, each containing 50,000 samples.

[a] ρ is a correlation coefficient between two unmeasured confounders.

eTable 4. Summary of estimated causal risk ratio when the BMI increases from 53.0 to 59.5 without age (measured confounder) in the outcome model

**Estimators**	**Simulation Settings**							
	**Using 25 Strong IVs and 25 Weak IVs**							
	**Bivariate Normal Distribution**		**Marginal Normal Distributions**				**Marginal t-distributions**	
			**With t-copula**		**With Clayton Copula**	**With Gumbel Copula**	**With Normal Copula**	
	$\rho^{a} = 0.5$	$\rho^{a} = -0.5$	$\rho^{a} = 0.5$	$\rho^{a} = -0.5$	$\rho^{a} = 0.5$	$\rho^{a} = 0.5$	$\rho^{a} = 0.5$	$\rho^{a} = -0.5$
$2SRI_{PR}$	1.132(0.000)	1.253(0.000)	1.153(0.000)	1.265(0.000)	1.104(0.000)	1.156(0.000)	1.167(0.000)	1.231(0.000)
$2SRI_{LL}$	1.387(0.000)	1.339(0.000)	1.412(0.000)	1.378(0.000)	1.482(0.000)	1.357(0.000)	1.384(0.000)	1.323(0.000)
LIML	1.065(0.250)	1.092(0.176)	1.066(0.214)	1.133(0.232)	1.156(0.143)	1.101(0.279)	1.134(0.310)	1.110(0.275)
2SLS	1.277(0.012)	1.270(0.019)	1.274(0.015)	1.264(0.022)	1.277(0.012)	1.274(0.014)	1.272(0.019)	1.265(0.024)

$2SPS_{PR}$	1.166(0.048)	1.263(0.057)	1.180(0.051)	1.275(0.054)	1.158(0.046)	1.177(0.052)	1.190(0.059)	1.241(0.062)
$2SPS_{LL}$	2.776(0.422)	2.602(0.544)	2.684(0.443)	2.466(0.546)	2.759(0.396)	2.696(0.461)	2.685(0.567)	2.524(0.611)
IVW_{LI}	1.123 (0.000)	1.123 (0.000)	1.123 (0.000)	1.123 (0.000)	1.123 (0.000)	1.123 (0.000)	1.123 (0.000)	1.123 (0.000)
IVW_{LL}	1.916 (0.288)	2.342 (0.457)	1.843 (0.322)	2.180 (0.458)	1.814 (0.280)	1.927 (0.339)	2.132 (0.449)	2.321 (0.527)

IV, instrumental variable; IVW_{LI}, inverse-variance weighted estimator with linear construction; IVW_{LL}, inverse-variance weighted estimator with non-linear construction; LIML, limited information maximum likelihood; 2SLS, two-stage least square; $2SPS_{PR}$, two-stage predictor substitution with probit link; $2SPS_{LL}$, two-stage predictor substitution with log link; $2SRI_{PR}$, two-stage residual inclusion with probit link; $2SRI_{LL}$, two-stage residual inclusion with log link.

The sample size n is 100,000, and the simulation is iterated 200 times. The true causal risk ratio is approximately 5.44. The mean and empirical standard error (ESE) represented as “mean (ESE)” in the table are summarized. The analyses utilized 50 out of the true 500 SNPs. For the WIV methods, the datasets were split into two subsets, each containing 50,000 samples.

[a] ρ is a correlation coefficient between two unmeasured confounders.

eTable 4 (continued). Summary of estimated causal risk ratio when the BMI increases from 53.0 to 59.5 without age (measured confounder) in the outcome model

**Estimators**	**Simulation Settings**							
	**Using Only 50 Weak IVs**							
	**Bivariate Normal Distribution**		**Marginal Normal Distributions**				**Marginal t-distributions**	
			**With t-copula**		**With Clayton Copula**	**With Gumbel Copula**	**With Normal Copula**	
	$\boldsymbol{\rho}^{\mathrm{a}} = \mathbf{0.5}$	$\boldsymbol{\rho}^{\mathrm{a}} = -\mathbf{0.5}$	$\boldsymbol{\rho}^{\mathrm{a}} = \mathbf{0.5}$	$\boldsymbol{\rho}^{\mathrm{a}} = -\mathbf{0.5}$	$\boldsymbol{\rho}^{\mathrm{a}} = \mathbf{0.5}$	$\boldsymbol{\rho}^{\mathrm{a}} = \mathbf{0.5}$	$\boldsymbol{\rho}^{\mathrm{a}} = \mathbf{0.5}$	$\boldsymbol{\rho}^{\mathrm{a}} = -\mathbf{0.5}$
$2SRI_{PR}$	1.003(0.000)	1.233(0.000)	1.007(0.000)	1.241(0.000)	1.001(0.000)	1.015(0.000)	1.014(0.000)	1.185(0.000)
$2SRI_{LL}$	1.021(0.000)	1.441(0.000)	1.025(0.000)	1.432(0.000)	1.020(0.000)	1.040(0.000)	1.038(0.000)	1.270(0.000)
LIML	0.929(0.371)	1.010(0.268)	0.901(0.386)	1.050(0.183)	1.012(0.114)	0.957(0.317)	0.636(0.489)	0.776(0.470)
2SLS	1.321(0.011)	1.163(0.404)	1.319(0.013)	1.409(1.517)	1.327(0.010)	1.313(0.014)	1.317(0.023)	1.279(0.521)

$2SPS_{PR}$	1.013(0.022)	1.137(0.239)	1.017(0.034)	1.098(0.311)	1.006(0.022)	1.032(0.042)	1.032(0.056)	1.134(0.246)
$2SPS_{LL}$	7.835(2.892)	1.579(0.762)	7.305(2.606)	1.662(0.869)	10.143(3.684)	6.124(2.373)	8.158(4.568)	2.414(1.624)
IVW_{LI}	1.123 (0.000)	1.123 (0.000)	1.123 (0.000)	1.123 (0.000)	1.123 (0.000)	1.123 (0.000)	1.123 (0.000)	1.123 (0.000)
IVW_{LL}	1.272 (0.464)	1.328 (0.497)	1.208 (0.443)	1.215 (0.443)	1.174 (0.475)	1.256 (0.494)	1.404 (0.738)	1.494 (0.813)

IV, instrumental variable; IVW_{LI}, inverse-variance weighted estimator with linear construction; IVW_{LL}, inverse-variance weighted estimator with non-linear construction; LIML, limited information maximum likelihood; 2SLS, two-stage least square; $2SPS_{PR}$, two-stage predictor substitution with probit link; $2SPS_{LL}$, two-stage predictor substitution with log link; $2SRI_{PR}$, two-stage residual inclusion with probit link; $2SRI_{LL}$, two-stage residual inclusion with log link.

The sample size n is 100,000, and the simulation is iterated 200 times. The true causal risk ratio is approximately 5.44. The mean and empirical standard error (ESE) represented as "mean (ESE)" in the table are summarized. The analyses utilized 50 out of the true 500 SNPs. For the WIV methods, the datasets were split into two subsets, each containing 50,000 samples.

[a] ρ is a correlation coefficient between two unmeasured confounders.

eTable 5. Summary of estimated causal risk differences when the BMI increases from 18.5 to 25.0 with age (measured confounder) included in the outcome model

**Estimators**	**Simulation Settings**							
	**Using 25 Strong IVs and 25 Weak IVs**							
	**Bivariate Normal Distribution**		**Marginal Normal Distributions**				**Marginal t-distributions**	
			**With t-copula**		**With Clayton Copula**	**With Gumbel Copula**	**With Normal Copula**	
	$\rho^a = 0.5$	$\rho^a = -0.5$	$\rho^a = 0.5$	$\rho^a = -0.5$	$\rho^a = 0.5$	$\rho^a = 0.5$	$\rho^a = 0.5$	$\rho^a = -0.5$
$2SRI_{PR}$	0.008(0.000)	0.018(0.000)	0.009(0.000)	0.019(0.000)	0.006(0.000)	0.011(0.000)	0.013(0.000)	0.019(0.000)
$2SRI_{LL}$	0.001(0.000)	0.002(0.000)	0.001(0.000)	0.003(0.000)	0.001(0.000)	0.001(0.000)	0.002(0.000)	0.003(0.000)
LIML	-0.001(0.000)	0.000(0.000)	0.006(0.000)	0.008(0.000)	0.007(0.000)	0.015(0.000)	0.016(0.000)	0.000(0.000)
2SLS	0.169(0.028)	0.114(0.028)	0.160(0.030)	0.108(0.028)	0.175(0.027)	0.161(0.030)	0.153(0.035)	0.122(0.034)
$2SPS_{PR}$	0.015(0.000)	0.014(0.000)	0.016(0.000)	0.015(0.000)	0.015(0.000)	0.016(0.000)	0.016(0.000)	0.017(0.000)

$2SPS_{LL}$	0.019(0.000)	0.016(0.000)	0.020(0.000)	0.017(0.000)	0.020(0.000)	0.020(0.000)	0.019(0.000)	0.018(0.000)
IVW_{LI}	0.120 (0.028)	0.118 (0.027)	0.112 (0.029)	0.111 (0.027)	0.115 (0.028)	0.120 (0.032)	0.132 (0.035)	0.130 (0.033)
IVW_{LL}	8.681 (7.032)	24.054 (23.640)	7.425 (5.968)	20.009 (24.726)	6.568 (4.980)	9.144 (8.035)	16.295 (16.364)	26.804 (31.370)

IV, instrumental variable; IVW_{LI}, inverse-variance weighted estimator with linear construction; IVW_{LL}, inverse-variance weighted estimator with non-linear construction; LIML, limited information maximum likelihood; 2SLS, two-stage least square; $2SPS_{PR}$, two-stage predictor substitution with probit link; $2SPS_{LL}$, two-stage predictor substitution with log link; $2SRI_{PR}$, two-stage residual inclusion with probit link; $2SRI_{LL}$, two-stage residual inclusion with log link.

The sample size n is 100,000, and the simulation is iterated 200 times. The true causal risk differences is approximately 0.014. The mean and empirical standard error (ESE) represented as "mean (ESE)" in the table are summarized. The analyses utilized 50 out of the true 500 SNPs. For the WIV methods, the datasets were split into two subsets, each containing 50,000 samples.

[a] ρ is a correlation coefficient between two unmeasured confounders.

eTable 5 (continued). Summary of estimated causal risk differences when the BMI increases from 18.5 to 25.0 with age (measured confounder) included in the outcome model

**Estimators**	**Simulation Settings**							
	**Using Only 50 Weak IVs**							
	**Bivariate Normal Distribution**		**Marginal Normal Distributions**				**Marginal t-distributions**	
			**With t-copula**		**With Clayton Copula**	**With Gumbel Copula**	**With Normal Copula**	
	$\rho^a = 0.5$	$\rho^a = -0.5$	$\rho^a = 0.5$	$\rho^a = -0.5$	$\rho^a = 0.5$	$\rho^a = 0.5$	$\rho^a = 0.5$	$\rho^a = -0.5$
$2SRI_{PR}$	0.000(0.000)	-0.078(0.000)	0.001(0.000)	-0.081(0.000)	0.000(0.000)	0.002(0.000)	0.004(0.000)	-0.075(0.000)
$2SRI_{LL}$	0.000(0.000)	-0.081(0.000)	0.000(0.000)	-0.096(0.000)	0.000(0.000)	0.000(0.000)	-0.003(0.000)	-0.088(0.000)
LIML	-0.027(0.000)	-0.016(0.000)	-0.021(0.000)	-0.016(0.000)	0.000(0.000)	0.003(0.000)	-0.002(0.000)	-0.003(0.000)
2SLS	0.323(0.071)	-0.046(0.063)	0.302(0.065)	-0.045(0.077)	0.405(0.071)	0.261(0.068)	0.282(0.110)	-0.036(0.106)

$2SPS_{PR}$	0.002(0.000)	-0.079(0.000)	0.002(0.000)	-0.085(0.000)	0.001(0.000)	0.005(0.000)	0.005(0.000)	-0.075(0.000)
$2SPS_{LL}$	0.007(0.000)	-0.649(0.012)	0.007(0.000)	-0.678(0.008)	0.004(0.000)	0.011(0.000)	0.009(0.000)	-1.228(0.017)
IVW_{LI}	0.043 (0.071)	0.038 (0.050)	0.033 (0.061)	0.026 (0.057)	0.031 (0.083)	0.034 (0.069)	0.061 (0.103)	0.070 (0.095)
IVW_{LL}	4.342 (10.916)	3.389 (8.918)	3.229 (12.451)	5.483 (27.665)	4.139 (14.376)	2.586 (5.892)	33.499 (124.535)	123.171 (585.361)

IV, instrumental variable; IVW_{LI}, inverse-variance weighted estimator with linear construction; IVW_{LL}, inverse-variance weighted estimator with non-linear construction; LIML, limited information maximum likelihood; 2SLS, two-stage least square; $2SPS_{PR}$, two-stage predictor substitution with probit link; $2SPS_{LL}$, two-stage predictor substitution with log link; $2SRI_{PR}$, two-stage residual inclusion with probit link; $2SRI_{LL}$, two-stage residual inclusion with log link.

The sample size n is 100,000, and the simulation is iterated 200 times. The true causal risk differences is approximately 0.014. The mean and empirical standard error (ESE) represented as “mean (ESE)” in the table are summarized. The analyses utilized 50 out of the true 500 SNPs. For the WIV methods, the datasets were split into two subsets, each containing 50,000 samples.

[b] ρ is a correlation coefficient between two unmeasured confounders.

eTable 6. Summary of estimated causal risk differences when the BMI increases from 18.5 to 25.0 without age (measured confounder) in the outcome model

Estimators	Simulation Settings							
	Using 25 Strong IVs and 25 Weak IVs							
	Bivariate Normal Distribution		Marginal Normal Distributions				Marginal t-distributions	
			With t-copula		With Clayton Copula	With Gumbel Copula	With Normal Copula	
	$\rho^a = 0.5$	$\rho^a = -0.5$	$\rho^a = 0.5$	$\rho^a = -0.5$	$\rho^a = 0.5$	$\rho^a = 0.5$	$\rho^a = 0.5$	$\rho^a = -0.5$
$2SRI_{PR}$	0.005(0.000)	0.016(0.000)	0.006(0.000)	0.017(0.000)	0.002(0.000)	0.008(0.000)	0.007(0.000)	0.017(0.000)
$2SRI_{LL}$	0.000(0.000)	0.000(0.000)	0.000(0.000)	0.001(0.000)	0.001(0.000)	0.000(0.000)	0.000(0.000)	0.001(0.000)
LIML	0.003(0.026)	0.005(0.016)	0.003(0.028)	0.006(0.031)	0.007(0.021)	0.004(0.037)	0.012(0.020)	0.008(0.028)
2SLS	0.179(0.027)	0.126(0.028)	0.170(0.029)	0.119(0.029)	0.184(0.027)	0.173(0.030)	0.164(0.035)	0.134(0.035)

$2SPS_{PR}$	0.016(0.005)	0.015(0.005)	0.017(0.006)	0.016(0.005)	0.016(0.006)	0.017(0.006)	0.017(0.007)	0.017(0.006)
$2SPS_{LL}$	0.020(0.003)	0.017(0.003)	0.021(0.004)	0.017(0.003)	0.021(0.003)	0.021(0.004)	0.020(0.004)	0.019(0.004)
IVW_{LI}	0.113 (0.027)	0.112 (0.027)	0.104 (0.030)	0.103 (0.027)	0.107 (0.029)	0.113 (0.031)	0.125 (0.036)	0.122 (0.033)
IVW_{LL}	7.005 (5.331)	19.698 (17.383)	6.173 (5.640)	15.269 (18.293)	5.427 (4.388)	7.664 (6.715)	13.534 (14.688)	21.175 (26.807)

IV, instrumental variable; IVW_{LI}, inverse-variance weighted estimator with linear construction; IVW_{LL}, inverse-variance weighted estimator with non-linear construction; LIML, limited information maximum likelihood; 2SLS, two-stage least square; $2SPS_{PR}$, two-stage predictor substitution with probit link; $2SPS_{LL}$, two-stage predictor substitution with log link; $2SRI_{PR}$, two-stage residual inclusion with probit link; $2SRI_{LL}$, two-stage residual inclusion with log link.

The sample size n is 100,000, and the simulation is iterated 200 times. The true causal risk differences is approximately 0.014. The mean and empirical standard error (ESE) represented as "mean (ESE)" in the table are summarized. The analyses utilized 50 out of the true 500 SNPs. For the WIV methods, the datasets were split into two subsets, each containing 50,000 samples.

[b] ρ is a correlation coefficient between two unmeasured confounders.

eTable 6 (continued). Summary of estimated causal risk differences when the BMI increases from 18.5 to 25.0 without age (measured confounder) in the outcome model

Estimators	Simulation Settings							
	Using Only 50 Weak IVs							
	Bivariate Normal Distribution		Marginal Normal Distributions				Marginal t-distributions	
			With t-copula		With Clayton Copula	With Gumbel Copula	With Normal Copula	
	$\rho^{a} = 0.5$	$\rho^{a} = -0.5$	$\rho^{a} = 0.5$	$\rho^{a} = -0.5$	$\rho^{a} = 0.5$	$\rho^{a} = 0.5$	$\rho^{a} = 0.5$	$\rho^{a} = -0.5$
$2SRI_{PR}$	0.000(0.000)	0.005(0.000)	0.000(0.000)	-0.006(0.000)	0.000(0.000)	0.001(0.000)	0.000(0.000)	0.003(0.000)
$2SRI_{LL}$	0.000(0.000)	-0.010(0.000)	0.000(0.000)	-0.023(0.000)	0.000(0.000)	0.000(0.000)	0.000(0.000)	-0.013(0.000)
LIML	-0.016(0.056)	-0.007(0.050)	-0.022(0.059)	0.001(0.026)	-0.003(0.022)	-0.013(0.050)	-0.052(0.080)	-0.021(0.063)
2SLS	0.354(0.065)	0.048(0.060)	0.338(0.062)	0.050(0.074)	0.413(0.068)	0.309(0.065)	0.332(0.094)	0.103(0.094)

$2SPS_{PR}$	0.001(0.002)	0.004(0.039)	0.002(0.003)	-0.006(0.056)	0.001(0.002)	0.003(0.004)	0.003(0.005)	0.003(0.046)
$2SPS_{LL}$	0.006(0.003)	-0.007(0.089)	0.006(0.004)	-0.045(0.224)	0.004(0.003)	0.008(0.004)	0.007(0.005)	-0.028(0.257)
IVW_{LI}	0.032 (0.062)	0.029 (0.049)	0.022 (0.062)	0.017 (0.051)	0.014 (0.075)	0.027 (0.068)	0.036 (0.085)	0.041 (0.075)
IVW_{LL}	2.082 (4.268)	2.747 (6.267)	1.717 (4.991)	1.644 (4.376)	1.716 (4.674)	2.415 (7.889)	8.073 (31.646)	11.971 (40.186)

IV, instrumental variable; IVW_{LI}, inverse-variance weighted estimator with linear construction; IVW_{LL}, inverse-variance weighted estimator with non-linear construction; LIML, limited information maximum likelihood; 2SLS, two-stage least square; $2SPS_{PR}$, two-stage predictor substitution with probit link; $2SPS_{LL}$, two-stage predictor substitution with log link; $2SRI_{PR}$, two-stage residual inclusion with probit link; $2SRI_{LL}$, two-stage residual inclusion with log link.

The sample size n is 100,000, and the simulation is iterated 200 times. The true causal risk differences is approximately 0.014. The mean and empirical standard error (ESE) represented as “mean (ESE)” in the table are summarized. The analyses utilized 50 out of the true 500 SNPs. For the WIV methods, the datasets were split into two subsets, each containing 50,000 samples.

[b] ρ is a correlation coefficient between two unmeasured confounders.

eTable 7. Summary of estimated causal risk differences when the BMI increases from 30.5 to 37.0 with age (measured confounder) included in the outcome model

Estimators	Simulation Settings							
	Using 25 Strong IVs and 25 Weak IVs							
	Bivariate Normal Distribution		Marginal Normal Distributions				Marginal t-distributions	
			With t-copula		With Clayton Copula	With Gumbel Copula	With Normal Copula	
	$\rho^a = 0.5$	$\rho^a = -0.5$	$\rho^a = 0.5$	$\rho^a = -0.5$	$\rho^a = 0.5$	$\rho^a = 0.5$	$\rho^a = 0.5$	$\rho^a = -0.5$
$2SRI_{PR}$	0.119(0.000)	0.091(0.000)	0.115(0.000)	0.087(0.000)	0.118(0.000)	0.118(0.000)	0.114(0.000)	0.097(0.000)
$2SRI_{LL}$	0.031(0.000)	0.037(0.000)	0.031(0.000)	0.035(0.000)	0.031(0.000)	0.033(0.000)	0.034(0.000)	0.036(0.000)
LIML	0.011(0.000)	0.086(0.000)	0.016(0.000)	0.076(0.000)	0.206(0.000)	0.108(0.000)	0.107(0.000)	0.183(0.000)
2SLS	0.169(0.028)	0.114(0.028)	0.160(0.030)	0.108(0.028)	0.175(0.027)	0.161(0.030)	0.153(0.035)	0.122(0.034)

$2SPS_{PR}$	0.129(0.000)	0.087(0.000)	0.124(0.000)	0.083(0.000)	0.135(0.000)	0.124(0.000)	0.118(0.000)	0.094(0.000)
$2SPS_{LL}$	0.112(0.000)	0.075(0.000)	0.107(0.000)	0.073(0.000)	0.116(0.000)	0.107(0.000)	0.102(0.000)	0.082(0.000)
IVW_{LI}	0.120 (0.028)	0.118 (0.027)	0.112 (0.029)	0.111 (0.027)	0.115 (0.028)	0.120 (0.032)	0.132 (0.035)	0.130 (0.033)
IVW_{LL}	39.052 (46.042)	173.228 (270.400)	31.365 (36.971)	144.042 (295.376)	26.084 (28.352)	43.057 (53.247)	101.162 (150.184)	216.835 (385.943)

IV, instrumental variable; IVW_{LI}, inverse-variance weighted estimator with linear construction; IVW_{LL}, inverse-variance weighted estimator with non-linear construction; LIML, limited information maximum likelihood; 2SLS, two-stage least square; $2SPS_{PR}$, two-stage predictor substitution with probit link; $2SPS_{LL}$, two-stage predictor substitution with log link; $2SRI_{PR}$, two-stage residual inclusion with probit link; $2SRI_{LL}$, two-stage residual inclusion with log link.

The sample size n is 100,000, and the simulation is iterated 200 times. The true causal risk differences is approximately 0.108. The mean and empirical standard error (ESE) represented as "mean (ESE)" in the table are summarized. The analyses utilized 50 out of the true 500 SNPs. For the WIV methods, the datasets were split into two subsets, each containing 50,000 samples.

[a] ρ is a correlation coefficient between two unmeasured confounders.

eTable 7 (continued). Summary of estimated causal risk differences when the BMI increases from 30.5 to 37.0 with age (measured confounder) included in the outcome model

Estimators	Simulation Settings							
	Using Only 50 Weak IVs							
	Bivariate Normal Distribution		Marginal Normal Distributions				Marginal t-distributions	
			With t-copula		With Clayton Copula	With Gumbel Copula	With Normal Copula	
	$\rho^a = 0.5$	$\rho^a = -0.5$	$\rho^a = 0.5$	$\rho^a = -0.5$	$\rho^a = 0.5$	$\rho^a = 0.5$	$\rho^a = 0.5$	$\rho^a = -0.5$
$2SRI_{PR}$	0.148(0.000)	-0.055(0.000)	0.146(0.000)	-0.052(0.000)	0.112(0.000)	0.150(0.000)	0.150(0.000)	-0.054(0.000)
$2SRI_{LL}$	0.027(0.000)	-0.033(0.000)	0.027(0.000)	-0.037(0.000)	0.022(0.000)	0.033(0.000)	0.033(0.000)	-0.036(0.000)
LIML	0.050(0.000)	0.037(0.000)	0.071(0.000)	0.040(0.000)	0.161(0.000)	0.161(0.000)	0.102(0.000)	0.189(0.000)
2SLS	0.323(0.071)	-0.046(0.063)	0.302(0.065)	-0.045(0.077)	0.405(0.071)	0.261(0.068)	0.282(0.110)	-0.036(0.106)

$2SPS_{PR}$	0.188(0.000)	-0.057(0.000)	0.182(0.000)	-0.056(0.000)	0.217(0.000)	0.167(0.000)	0.166(0.000)	-0.055(0.000)
$2SPS_{LL}$	0.162(0.000)	-0.082(0.001)	0.156(0.000)	-0.088(0.001)	0.189(0.000)	0.143(0.000)	0.142(0.000)	-0.098(0.001)
IVW_{LI}	0.043 (0.071)	0.038 (0.050)	0.033 (0.061)	0.026 (0.057)	0.031 (0.083)	0.034 (0.069)	0.061 (0.103)	0.070 (0.095)
IVW_{LL}	26.090 (86.988)	17.610 (81.825)	22.702 (124.192)	58.459 (447.970)	30.127 (149.119)	11.668 (36.316)	665.363 (> 999)	> 999 (> 999)

IV, instrumental variable; IVW_{LI}, inverse-variance weighted estimator with linear construction; IVW_{LL}, inverse-variance weighted estimator with non-linear construction; LIML, limited information maximum likelihood; 2SLS, two-stage least square; $2SPS_{PR}$, two-stage predictor substitution with probit link; $2SPS_{LL}$, two-stage predictor substitution with log link; $2SRI_{PR}$, two-stage residual inclusion with probit link; $2SRI_{LL}$, two-stage residual inclusion with log link.

The sample size n is 100,000, and the simulation is iterated 200 times. The true causal risk differences is approximately 0.108. The mean and empirical standard error (ESE) represented as “mean (ESE)” in the table are summarized. The analyses utilized 50 out of the true 500 SNPs. For the WIV methods, the datasets were split into two subsets, each containing 50,000 samples.

[a] ρ is a correlation coefficient between two unmeasured confounders.

eTable 8. Summary of estimated causal risk differences when the BMI increases from 30.5 to 37.0 without age (measured confounder) in the outcome model

Estimators	Simulation Settings							
	Using 25 Strong IVs and 25 Weak IVs							
	Bivariate Normal Distribution		Marginal Normal Distributions				Marginal t-distributions	
			With t-copula		With Clayton Copula	With Gumbel Copula	With Normal Copula	
	$\rho^{a} = 0.5$	$\rho^{a} = -0.5$	$\rho^{a} = 0.5$	$\rho^{a} = -0.5$	$\rho^{a} = 0.5$	$\rho^{a} = 0.5$	$\rho^{a} = 0.5$	$\rho^{a} = -0.5$
$2SRI_{PR}$	0.118(0.000)	0.099(0.000)	0.115(0.000)	0.094(0.000)	0.109(0.000)	0.121(0.000)	0.111(0.000)	0.105(0.000)
$2SRI_{LL}$	0.027(0.000)	0.032(0.000)	0.026(0.000)	0.031(0.000)	0.025(0.000)	0.029(0.000)	0.024(0.000)	0.033(0.000)
LIML	0.145(0.184)	0.229(0.179)	0.159(0.142)	0.143(0.151)	0.123(0.070)	0.107(0.159)	0.068(0.214)	0.180(0.274)
2SLS	0.179(0.027)	0.126(0.028)	0.170(0.029)	0.119(0.029)	0.184(0.027)	0.173(0.030)	0.164(0.035)	0.134(0.035)

$2SPS_{PR}$	0.138(0.015)	0.098(0.017)	0.132(0.017)	0.093(0.018)	0.142(0.016)	0.134(0.018)	0.127(0.021)	0.105(0.021)
$2SPS_{LL}$	0.129(0.014)	0.092(0.015)	0.124(0.016)	0.088(0.016)	0.133(0.014)	0.125(0.016)	0.119(0.019)	0.099(0.019)
IVW_{LI}	0.113 (0.027)	0.112 (0.027)	0.104 (0.030)	0.103 (0.027)	0.107 (0.029)	0.113 (0.031)	0.125 (0.036)	0.122 (0.033)
IVW_{LL}	28.528 (31.523)	127.328 (160.413)	25.080 (33.564)	97.671 (184.498)	20.280 (24.236)	33.566 (42.521)	79.420 (135.798)	158.700 (326.860)

IV, instrumental variable; IVW_{LI}, inverse-variance weighted estimator with linear construction; IVW_{LL}, inverse-variance weighted estimator with non-linear construction; LIML, limited information maximum likelihood; 2SLS, two-stage least square; $2SPS_{PR}$, two-stage predictor substitution with probit link; $2SPS_{LL}$, two-stage predictor substitution with log link; $2SRI_{PR}$, two-stage residual inclusion with probit link; $2SRI_{LL}$, two-stage residual inclusion with log link.

The sample size n is 100,000, and the simulation is iterated 200 times. The true causal risk differences is approximately 0.108. The mean and empirical standard error (ESE) represented as “mean (ESE)” in the table are summarized. The analyses utilized 50 out of the true 500 SNPs. For the WIV methods, the datasets were split into two subsets, each containing 50,000 samples.

[a] ρ is a correlation coefficient between two unmeasured confounders.

eTable 8 (continued). Summary of estimated causal risk differences when the BMI increases from 30.5 to 37.0 without age (measured confounder) in the outcome model

Estimators	Simulation Settings							
	Using Only 50 Weak IVs							
	Bivariate Normal Distribution		Marginal Normal Distributions				Marginal t-distributions	
			With t-copula		With Clayton Copula	With Gumbel Copula	With Normal Copula	
	$\rho^a = 0.5$	$\rho^a = -0.5$	$\rho^a = 0.5$	$\rho^a = -0.5$	$\rho^a = 0.5$	$\rho^a = 0.5$	$\rho^a = 0.5$	$\rho^a = -0.5$
$2SRI_{PR}$	0.150(0.000)	0.038(0.000)	0.149(0.000)	0.037(0.000)	0.105(0.000)	0.160(0.000)	0.157(0.000)	0.074(0.000)
$2SRI_{LL}$	0.026(0.000)	0.011(0.000)	0.025(0.000)	0.009(0.000)	0.019(0.000)	0.031(0.000)	0.029(0.000)	0.021(0.000)
LIML	0.049(0.181)	0.206(0.186)	0.043(0.186)	0.219(0.148)	0.114(0.056)	0.121(0.179)	-0.106(0.281)	0.014(0.391)
2SLS	0.354(0.065)	0.048(0.060)	0.338(0.062)	0.050(0.074)	0.413(0.068)	0.309(0.065)	0.332(0.094)	0.103(0.094)

$2SPS_{PR}$	0.210(0.021)	0.038(0.051)	0.203(0.022)	0.036(0.066)	0.231(0.020)	0.194(0.024)	0.196(0.034)	0.074(0.071)
$2SPS_{LL}$	0.200(0.022)	0.035(0.051)	0.193(0.023)	0.032(0.068)	0.222(0.022)	0.184(0.024)	0.187(0.035)	0.069(0.073)
IVW_{LI}	0.032 (0.062)	0.029 (0.049)	0.022 (0.062)	0.017 (0.051)	0.014 (0.075)	0.027 (0.068)	0.036 (0.085)	0.041 (0.075)
IVW_{LL}	8.327 (20.940)	12.779 (39.657)	7.700 (31.190)	6.769 (26.513)	7.490 (26.960)	13.083 (66.339)	83.902 (504.888)	137.990 (607.608)

IV, instrumental variable; IVW_{LI}, inverse-variance weighted estimator with linear construction; IVW_{LL}, inverse-variance weighted estimator with non-linear construction; LIML, limited information maximum likelihood; 2SLS, two-stage least square; $2SPS_{PR}$, two-stage predictor substitution with probit link; $2SPS_{LL}$, two-stage predictor substitution with log link; $2SRI_{PR}$, two-stage residual inclusion with probit link; $2SRI_{LL}$, two-stage residual inclusion with log link.

The sample size n is 100,000, and the simulation is iterated 200 times. The true causal risk differences is approximately 2.96. The mean and empirical standard error (ESE) represented as “mean (ESE)” in the table are summarized. The analyses utilized 50 out of the true 500 SNPs. For the WIV methods, the datasets were split into two subsets, each containing 50,000 samples.

[a] ρ is a correlation coefficient between two unmeasured confounders.

eTable 9. Summary of estimated causal risk differences when the BMI increases from 41.0 to 47.5 with age (measured confounder) included in the outcome model

**Estimators**	**Simulation Settings**							
	**Using 25 Strong IVs and 25 Weak IVs**							
	**Bivariate Normal Distribution**		**Marginal Normal Distributions**				**Marginal t-distributions**	
			**With t-copula**		**With Clayton Copula**	**With Gumbel Copula**	**With Normal Copula**	
	$\boldsymbol{\rho}^{\mathrm{a}} = \mathbf{0.5}$	$\boldsymbol{\rho}^{\mathrm{a}} = -\mathbf{0.5}$	$\boldsymbol{\rho}^{\mathrm{a}} = \mathbf{0.5}$	$\boldsymbol{\rho}^{\mathrm{a}} = -\mathbf{0.5}$	$\boldsymbol{\rho}^{\mathrm{a}} = \mathbf{0.5}$	$\boldsymbol{\rho}^{\mathrm{a}} = \mathbf{0.5}$	$\boldsymbol{\rho}^{\mathrm{a}} = \mathbf{0.5}$	$\boldsymbol{\rho}^{\mathrm{a}} = -\mathbf{0.5}$
$2SRI_{PR}$	0.298(0.000)	0.184(0.000)	0.280(0.000)	0.174(0.000)	0.325(0.000)	0.270(0.000)	0.256(0.000)	0.195(0.000)
$2SRI_{LL}$	0.217(0.000)	0.158(0.000)	0.203(0.000)	0.147(0.000)	0.179(0.000)	0.218(0.000)	0.207(0.000)	0.164(0.000)
LIML	0.267(0.000)	0.147(0.000)	0.244(0.000)	0.157(0.000)	0.168(0.000)	0.233(0.000)	0.227(0.000)	0.320(0.000)
2SLS	0.169(0.028)	0.114(0.028)	0.160(0.030)	0.108(0.028)	0.175(0.027)	0.161(0.030)	0.153(0.035)	0.122(0.034)

2SPS$_{PR}$	0.263(0.000)	0.195(0.000)	0.251(0.000)	0.183(0.000)	0.267(0.000)	0.249(0.000)	0.242(0.000)	0.199(0.000)
2SPS$_{LL}$	0.553(0.001)	0.328(0.001)	0.507(0.001)	0.296(0.001)	0.573(0.001)	0.497(0.001)	0.491(0.001)	0.356(0.001)
IVW$_{LI}$	0.120 (0.028)	0.118 (0.027)	0.112 (0.029)	0.111 (0.027)	0.115 (0.028)	0.120 (0.032)	0.132 (0.035)	0.130 (0.033)
IVW$_{LL}$	154.192 (236.377)	> 999 (> 999)	116.845 (184.932)	921.974 (> 999)	91.574 (128.522)	177.639 (273.232)	541.650 (> 999)	> 999 (> 999)

IV, instrumental variable; IVW$_{LI}$, inverse-variance weighted estimator with linear construction; IVW$_{LL}$, inverse-variance weighted estimator with non-linear construction; LIML, limited information maximum likelihood; 2SLS, two-stage least square; 2SPS$_{PR}$, two-stage predictor substitution with probit link; 2SPS$_{LL}$, two-stage predictor substitution with log link; 2SRI$_{PR}$, two-stage residual inclusion with probit link; 2SRI$_{LL}$, two-stage residual inclusion with log link.

The sample size n is 100,000, and the simulation is iterated 200 times. The true causal risk differences is approximately 0.230. The mean and empirical standard error (ESE) represented as “mean (ESE)” in the table are summarized. The analyses utilized 50 out of the true 500 SNPs. For the WIV methods, the datasets were split into two subsets, each containing 50,000 samples.

[a] ρ is a correlation coefficient between two unmeasured confounders.

eTable 9 (continued). Summary of estimated causal risk differences when the BMI increases from 41.0 to 47.5 with age (measured confounder) included in the outcome model

Estimators	Simulation Settings							
	Using Only 50 Weak IVs							
	Bivariate Normal Distribution		Marginal Normal Distributions				Marginal t-distributions	
			With t-copula		With Clayton Copula	With Gumbel Copula	With Normal Copula	
	$\rho^{a} = 0.5$	$\rho^{a} = -0.5$	$\rho^{a} = 0.5$	$\rho^{a} = -0.5$	$\rho^{a} = 0.5$	$\rho^{a} = 0.5$	$\rho^{a} = 0.5$	$\rho^{a} = -0.5$
$2SRI_{PR}$	0.416(0.000)	-0.015(0.000)	0.416(0.000)	-0.003(0.000)	0.326(0.000)	0.395(0.000)	0.360(0.000)	0.007(0.000)
$2SRI_{LL}$	0.559(0.000)	0.000(0.000)	0.534(0.000)	0.008(0.000)	0.595(0.000)	0.462(0.000)	0.448(0.000)	0.025(0.000)
LIML	0.239(0.000)	0.125(0.000)	0.274(0.000)	0.124(0.000)	0.131(0.000)	0.311(0.000)	0.258(0.000)	0.258(0.000)
2SLS	0.323(0.071)	-0.046(0.063)	0.302(0.065)	-0.045(0.077)	0.405(0.071)	0.261(0.068)	0.282(0.110)	-0.036(0.106)

$2SPS_{PR}$	0.379(0.000)	-0.018(0.000)	0.383(0.000)	-0.006(0.000)	0.354(0.000)	0.359(0.000)	0.343(0.000)	0.009(0.000)
$2SPS_{LL}$	3.980(0.002)	-0.013(0.000)	3.281(0.002)	0.005(0.000)	8.226(0.003)	2.065(0.001)	4.016(0.002)	0.062(0.000)
IVW_{LI}	0.043 (0.071)	0.038 (0.050)	0.033 (0.061)	0.026 (0.057)	0.031 (0.083)	0.034 (0.069)	0.061 (0.103)	0.070 (0.095)
IVW_{LL}	139.652 (549.518)	91.322 (595.765)	149.057 (939.555)	586.546 (> 999)	200.293 (> 999)	48.684 (183.345)	> 999 (> 999)	> 999 (> 999)

IV, instrumental variable; IVW_{LI}, inverse-variance weighted estimator with linear construction; IVW_{LL}, inverse-variance weighted estimator with non-linear construction; LIML, limited information maximum likelihood; 2SLS, two-stage least square; $2SPS_{PR}$, two-stage predictor substitution with probit link; $2SPS_{LL}$, two-stage predictor substitution with log link; $2SRI_{PR}$, two-stage residual inclusion with probit link; $2SRI_{LL}$, two-stage residual inclusion with log link.

The sample size n is 100,000, and the simulation is iterated 200 times. The true causal risk differences is approximately 0.230. The mean and empirical standard error (ESE) represented as “mean (ESE)” in the table are summarized. The analyses utilized 50 out of the true 500 SNPs. For the WIV methods, the datasets were split into two subsets, each containing 50,000 samples.

[a] ρ is a correlation coefficient between two unmeasured confounders.

eTable 10. Summary of estimated causal risk differences when the BMI increases from 41.0 to 47.5 without age (measured confounder) in the outcome model

**Estimators**	**Simulation Settings**							
	**Using 25 Strong IVs and 25 Weak IVs**							
	**Bivariate Normal Distribution**		**Marginal Normal Distributions**				**Marginal t-distributions**	
			**With t-copula**		**With Clayton Copula**	**With Gumbel Copula**	**With Normal Copula**	
	$\rho^a = 0.5$	$\rho^a = -0.5$	$\rho^a = 0.5$	$\rho^a = -0.5$	$\rho^a = 0.5$	$\rho^a = 0.5$	$\rho^a = 0.5$	$\rho^a = -0.5$
$2SRI_{PR}$	0.331(0.000)	0.210(0.000)	0.311(0.000)	0.196(0.000)	0.372(0.000)	0.300(0.000)	0.298(0.000)	0.216(0.000)
$2SRI_{LL}$	0.244(0.000)	0.259(0.000)	0.229(0.000)	0.235(0.000)	0.199(0.000)	0.255(0.000)	0.239(0.000)	0.264(0.000)
LIML	0.194(0.139)	0.216(0.113)	0.237(0.160)	0.238(0.103)	0.240(0.121)	0.228(0.128)	0.208(0.105)	0.166(0.121)
2SLS	0.179(0.027)	0.126(0.028)	0.170(0.029)	0.119(0.029)	0.184(0.027)	0.173(0.030)	0.164(0.035)	0.134(0.035)

2SPS$_{PR}$	0.267(0.040)	0.213(0.057)	0.255(0.046)	0.198(0.059)	0.269(0.040)	0.258(0.046)	0.249(0.056)	0.215(0.064)
2SPS$_{LL}$	0.695(0.246)	0.465(0.231)	0.638(0.243)	0.413(0.227)	0.707(0.229)	0.653(0.261)	0.630(0.316)	0.487(0.291)
IVW$_{LI}$	0.113 (0.027)	0.112 (0.027)	0.104 (0.030)	0.103 (0.027)	0.107 (0.029)	0.113 (0.031)	0.125 (0.036)	0.122 (0.033)
IVW$_{LL}$	102.559 (148.397)	695.789 (> 999)	91.272 (160.298)	554.120 (> 999)	67.906 (> 999)	129.919 (214.721)	411.013 (980.822)	> 999 (> 999)

IV, instrumental variable; IVW$_{LI}$, inverse-variance weighted estimator with linear construction; IVW$_{LL}$, inverse-variance weighted estimator with non-linear construction; LIML, limited information maximum likelihood; 2SLS, two-stage least square; 2SPS$_{PR}$, two-stage predictor substitution with probit link; 2SPS$_{LL}$, two-stage predictor substitution with log link; 2SRI$_{PR}$, two-stage residual inclusion with probit link; 2SRI$_{LL}$, two-stage residual inclusion with log link.

The sample size n is 100,000, and the simulation is iterated 200 times. The true causal risk differences is approximately 0.230. The mean and empirical standard error (ESE) represented as “mean (ESE)” in the table are summarized. The analyses utilized 50 out of the true 500 SNPs. For the WIV methods, the datasets were split into two subsets, each containing 50,000 samples.

[a] ρ is a correlation coefficient between two unmeasured confounders.

eTable 10 (continued). Summary of estimated causal risk differences when the BMI increases from 41.0 to 47.5 without age (measured confounder) in the outcome model

**Estimators**	**Simulation Settings**							
	**Using Only 50 Weak IVs**							
	**Bivariate Normal Distribution**		**Marginal Normal Distributions**				**Marginal t-distributions**	
			**With t-copula**		**With Clayton Copula**	**With Gumbel Copula**	**With Normal Copula**	
	$\boldsymbol{\rho}^{\mathrm{a}} = \mathbf{0.5}$	$\boldsymbol{\rho}^{\mathrm{a}} = \mathbf{-0.5}$	$\boldsymbol{\rho}^{\mathrm{a}} = \mathbf{0.5}$	$\boldsymbol{\rho}^{\mathrm{a}} = \mathbf{-0.5}$	$\boldsymbol{\rho}^{\mathrm{a}} = \mathbf{0.5}$	$\boldsymbol{\rho}^{\mathrm{a}} = \mathbf{0.5}$	$\boldsymbol{\rho}^{\mathrm{a}} = \mathbf{0.5}$	$\boldsymbol{\rho}^{\mathrm{a}} = \mathbf{-0.5}$
$2SRI_{PR}$	0.406(0.000)	0.079(0.000)	0.417(0.000)	0.090(0.000)	0.312(0.000)	0.414(0.000)	0.391(0.000)	0.166(0.000)
$2SRI_{LL}$	0.606(0.000)	0.086(0.000)	0.595(0.000)	0.106(0.000)	0.623(0.000)	0.560(0.000)	0.546(0.000)	0.210(0.000)
LIML	0.235(0.196)	0.200(0.128)	0.199(0.193)	0.238(0.114)	0.288(0.186)	0.188(0.161)	0.058(0.157)	0.099(0.139)
2SLS	0.354(0.065)	0.048(0.060)	0.338(0.062)	0.050(0.074)	0.413(0.068)	0.309(0.065)	0.332(0.094)	0.103(0.094)

$2SPS_{PR}$	0.371(0.023)	0.080(0.093)	0.375(0.030)	0.092(0.104)	0.348(0.036)	0.368(0.033)	0.358(0.053)	0.166(0.133)
$2SPS_{LL}$	6.275(4.437)	0.158(0.260)	5.399(3.897)	0.194(0.294)	10.471(7.305)	3.955(3.316)	7.244(7.927)	0.617(1.057)
IVW_{LI}	0.032 (0.062)	0.029 (0.049)	0.022 (0.062)	0.017 (0.051)	0.014 (0.075)	0.027 (0.068)	0.036 (0.085)	0.041 (0.075)
IVW_{LL}	29.824 (86.409)	54.388 (208.334)	32.434 (160.705)	26.565 (135.320)	30.002 (129.721)	68.635 (446.267)	801.339 (> 999)	> 999 (> 999)

IV, instrumental variable; IVW_{LI}, inverse-variance weighted estimator with linear construction; IVW_{LL}, inverse-variance weighted estimator with non-linear construction; LIML, limited information maximum likelihood; 2SLS, two-stage least square; $2SPS_{PR}$, two-stage predictor substitution with probit link; $2SPS_{LL}$, two-stage predictor substitution with log link; $2SRI_{PR}$, two-stage residual inclusion with probit link; $2SRI_{LL}$, two-stage residual inclusion with log link.

The sample size n is 100,000, and the simulation is iterated 200 times. The true causal risk differences is approximately 0.230. The mean and empirical standard error (ESE) represented as "mean (ESE)" in the table are summarized. The analyses utilized 50 out of the true 500 SNPs. For the WIV methods, the datasets were split into two subsets, each containing 50,000 samples.

[a] ρ is a correlation coefficient between two unmeasured confounders.

eTable 11. Summary of estimated causal risk differences when the BMI increases from 53.0 to 59.5 with age (measured confounder) included in the outcome model

**Estimators**	**Simulation Settings**							
	**Using 25 Strong IVs and 25 Weak IVs**							
	**Bivariate Normal Distribution**		**Marginal Normal Distributions**				**Marginal t-distributions**	
			**With t-copula**		**With Clayton Copula**	**With Gumbel Copula**	**With Normal Copula**	
	$\boldsymbol{\rho}^{\mathrm{a}} = \mathbf{0.5}$	$\boldsymbol{\rho}^{\mathrm{a}} = -\mathbf{0.5}$	$\boldsymbol{\rho}^{\mathrm{a}} = \mathbf{0.5}$	$\boldsymbol{\rho}^{\mathrm{a}} = -\mathbf{0.5}$	$\boldsymbol{\rho}^{\mathrm{a}} = \mathbf{0.5}$	$\boldsymbol{\rho}^{\mathrm{a}} = \mathbf{0.5}$	$\boldsymbol{\rho}^{\mathrm{a}} = \mathbf{0.5}$	$\boldsymbol{\rho}^{\mathrm{a}} = -\mathbf{0.5}$
$2SRI_{PR}$	0.129(0.000)	0.164(0.000)	0.138(0.000)	0.162(0.000)	0.115(0.000)	0.140(0.000)	0.142(0.000)	0.155(0.000)
$2SRI_{LL}$	0.240(0.000)	0.216(0.000)	0.241(0.000)	0.209(0.000)	0.242(0.000)	0.234(0.000)	0.217(0.000)	0.207(0.000)
LIML	0.069(0.000)	0.100(0.000)	0.088(0.000)	0.111(0.000)	0.087(0.000)	0.151(0.000)	0.149(0.000)	0.002(0.000)
2SLS	0.169(0.028)	0.114(0.028)	0.160(0.030)	0.108(0.028)	0.175(0.027)	0.161(0.030)	0.153(0.035)	0.122(0.034)

$2SPS_{PR}$	0.136(0.000)	0.168(0.000)	0.142(0.000)	0.167(0.000)	0.130(0.000)	0.142(0.000)	0.144(0.000)	0.157(0.000)
$2SPS_{LL}$	3.734(0.005)	2.023(0.005)	3.224(0.005)	1.682(0.005)	3.761(0.004)	3.122(0.005)	3.355(0.005)	2.231(0.005)
IVW_{LI}	0.120 (0.028)	0.118 (0.027)	0.112 (0.029)	0.111 (0.027)	0.115 (0.028)	0.120 (0.032)	0.132 (0.035)	0.130 (0.033)
IVW_{LL}	785.510 (> 999)	> 999 (> 999)	556.968 (> 999)	> 999 (> 999)	404.871 (723.707)	949.888 (> 999)	> 999 (> 999)	> 999 (> 999)

IV, instrumental variable; IVW_{LI}, inverse-variance weighted estimator with linear construction; IVW_{LL}, inverse-variance weighted estimator with non-linear construction; LIML, limited information maximum likelihood; 2SLS, two-stage least square; $2SPS_{PR}$, two-stage predictor substitution with probit link; $2SPS_{LL}$, two-stage predictor substitution with log link; $2SRI_{PR}$, two-stage residual inclusion with probit link; $2SRI_{LL}$, two-stage residual inclusion with log link.

The sample size n is 100,000, and the simulation is iterated 200 times. The true causal risk differences is approximately 0.173. The mean and empirical standard error (ESE) represented as “mean (ESE)” in the table are summarized. The analyses utilized 50 out of the true 500 SNPs. For the WIV methods, the datasets were split into two subsets, each containing 50,000 samples.

[b] ρ is a correlation coefficient between two unmeasured confounders.

eTable 11 (continued). Summary of estimated causal risk differences when the BMI increases from 53.0 to 59.5 with age (measured confounder) included in the outcome model

**Estimators**	**Simulation Settings**							
	**Using Only 50 Weak IVs**							
	**Bivariate Normal Distribution**		**Marginal Normal Distributions**				**Marginal t-distributions**	
			**With t-copula**		**With Clayton Copula**	**With Gumbel Copula**	**With Normal Copula**	
	$\rho^{a}=0.5$	$\rho^{a}=-0.5$	$\rho^{a}=0.5$	$\rho^{a}=-0.5$	$\rho^{a}=0.5$	$\rho^{a}=0.5$	$\rho^{a}=0.5$	$\rho^{a}=-0.5$
$2SRI_{PR}$	0.011(0.000)	0.002(0.000)	0.022(0.000)	0.014(0.000)	0.001(0.000)	0.041(0.000)	0.038(0.000)	0.016(0.000)
$2SRI_{LL}$	0.044(0.000)	0.011(0.000)	0.058(0.000)	0.018(0.000)	0.029(0.000)	0.095(0.000)	0.071(0.000)	0.023(0.000)
LIML	0.043(0.000)	0.055(0.000)	0.035(0.000)	0.049(0.000)	0.003(0.000)	0.042(0.000)	0.066(0.000)	0.010(0.000)
2SLS	0.323(0.071)	-0.046(0.063)	0.302(0.065)	-0.045(0.077)	0.405(0.071)	0.261(0.068)	0.282(0.110)	-0.036(0.106)

$2SPS_{PR}$	0.021(0.000)	0.001(0.000)	0.026(0.000)	0.012(0.000)	0.005(0.000)	0.054(0.000)	0.042(0.000)	0.016(0.000)
$2SPS_{LL}$	226.335(0.084)	0.039(0.000)	177.872(0.076)	0.146(0.001)	939.782(0.806)	74.530(0.051)	446.785(0.266)	1.149(0.007)
IVW_{LI}	0.043 (0.071)	0.038 (0.050)	0.033 (0.061)	0.026 (0.057)	0.031 (0.083)	0.034 (0.069)	0.061 (0.103)	0.070 (0.095)
IVW_{LL}	> 999 (> 999)	716.320 (> 999)	> 999 (> 999)	> 999 (> 999)	> 999 (> 999)	271.348 (> 999)	> 999 (> 999)	> 999 (> 999)

IV, instrumental variable; IVW_{LI}, inverse-variance weighted estimator with linear construction; IVW_{LL}, inverse-variance weighted estimator with non-linear construction; LIML, limited information maximum likelihood; 2SLS, two-stage least square; $2SPS_{PR}$, two-stage predictor substitution with probit link; $2SPS_{LL}$, two-stage predictor substitution with log link; $2SRI_{PR}$, two-stage residual inclusion with probit link; $2SRI_{LL}$, two-stage residual inclusion with log link.

The sample size n is 100,000, and the simulation is iterated 200 times. The true causal risk differences is approximately 0.173. The mean and empirical standard error (ESE) represented as "mean (ESE)" in the table are summarized. The analyses utilized 50 out of the true 500 SNPs. For the WIV methods, the datasets were split into two subsets, each containing 50,000 samples.

[b] ρ is a correlation coefficient between two unmeasured confounders.

eTable 12. Summary of estimated causal risk differences when the BMI increases from 53.0 to 59.5 without age (measured confounder) in the outcome model

Estimators	Simulation Settings							
	Using 25 Strong IVs and 25 Weak IVs							
	Bivariate Normal Distribution		Marginal Normal Distributions				Marginal t-distributions	
			With t-copula		With Clayton Copula	With Gumbel Copula	With Normal Copula	
	$\rho^{a} = 0.5$	$\rho^{a} = -0.5$	$\rho^{a} = 0.5$	$\rho^{a} = -0.5$	$\rho^{a} = 0.5$	$\rho^{a} = 0.5$	$\rho^{a} = 0.5$	$\rho^{a} = -0.5$
$2SRI_{PR}$	0.112(0.000)	0.164(0.000)	0.125(0.000)	0.163(0.000)	0.092(0.000)	0.126(0.000)	0.132(0.000)	0.154(0.000)
$2SRI_{LL}$	0.240(0.000)	0.216(0.000)	0.243(0.000)	0.223(0.000)	0.257(0.000)	0.228(0.000)	0.229(0.000)	0.206(0.000)
LIML	0.074(0.076)	0.073(0.077)	0.058(0.075)	0.105(0.074)	0.112(0.072)	0.105(0.070)	0.130(0.055)	0.101(0.072)
2SLS	0.179(0.027)	0.126(0.028)	0.170(0.029)	0.119(0.029)	0.184(0.027)	0.173(0.030)	0.164(0.035)	0.134(0.035)

$2SPS_{PR}$	0.129(0.025)	0.165(0.020)	0.135(0.024)	0.164(0.021)	0.124(0.024)	0.133(0.026)	0.137(0.028)	0.154(0.027)
$2SPS_{LL}$	5.127(3.531)	3.351(3.021)	4.491(3.042)	2.788(2.958)	5.067(2.872)	4.695(3.485)	4.842(4.925)	3.595(4.250)
IVW_{LI}	0.113 (0.027)	0.112 (0.027)	0.104 (0.030)	0.103 (0.027)	0.107 (0.029)	0.113 (0.031)	0.125 (0.036)	0.122 (0.033)
IVW_{LL}	467.062 (877.621)	> 999 (> 999)	427.017 (966.634)	> 999 (> 999)	286.922 (619.528)	648.825 (> 999)	> 999 (> 999)	> 999 (> 999)

IV, instrumental variable; IVW_{LI}, inverse-variance weighted estimator with linear construction; IVW_{LL}, inverse-variance weighted estimator with non-linear construction; LIML, limited information maximum likelihood; 2SLS, two-stage least square; $2SPS_{PR}$, two-stage predictor substitution with probit link; $2SPS_{LL}$, two-stage predictor substitution with log link; $2SRI_{PR}$, two-stage residual inclusion with probit link; $2SRI_{LL}$, two-stage residual inclusion with log link.

The sample size n is 100,000, and the simulation is iterated 200 times. The true causal risk differences is approximately 0.173. The mean and empirical standard error (ESE) represented as "mean (ESE)" in the table are summarized. The analyses utilized 50 out of the true 500 SNPs. For the WIV methods, the datasets were split into two subsets, each containing 50,000 samples.

[b] ρ is a correlation coefficient between two unmeasured confounders.

eTable 12 (continued). Summary of estimated causal risk differences when the BMI increases from 53.0 to 59.5 without age (measured confounder) in the outcome model

Estimators	Simulation Settings							
	Using Only 50 Weak IVs							
	Bivariate Normal Distribution		Marginal Normal Distributions				Marginal t-distributions	
			With t-copula		With Clayton Copula	With Gumbel Copula	With Normal Copula	
	$\rho^a = 0.5$	$\rho^a = -0.5$	$\rho^a = 0.5$	$\rho^a = -0.5$	$\rho^a = 0.5$	$\rho^a = 0.5$	$\rho^a = 0.5$	$\rho^a = -0.5$
$2SRI_{PR}$	0.003(0.000)	0.077(0.000)	0.007(0.000)	0.086(0.000)	0.001(0.000)	0.014(0.000)	0.013(0.000)	0.098(0.000)
$2SRI_{LL}$	0.021(0.000)	0.103(0.000)	0.024(0.000)	0.121(0.000)	0.019(0.000)	0.038(0.000)	0.036(0.000)	0.127(0.000)
LIML	0.046(0.063)	0.038(0.062)	0.042(0.066)	0.047(0.068)	0.019(0.045)	0.035(0.060)	0.023(0.057)	0.026(0.054)
2SLS	0.354(0.065)	0.048(0.060)	0.338(0.062)	0.050(0.074)	0.413(0.068)	0.309(0.065)	0.332(0.094)	0.103(0.094)

2SPS$_{PR}$	0.013(0.020)	0.077(0.069)	0.016(0.024)	0.087(0.074)	0.005(0.017)	0.028(0.033)	0.026(0.039)	0.098(0.066)
2SPS$_{LL}$	461.656(674.213)	1.139(3.277)	350.800(619.124)	1.505(3.987)	> 999(> 999)	209.738(528.750)	945.123(> 999)	14.314(51.375)
IVW$_{LI}$	0.032 (0.062)	0.029 (0.049)	0.022 (0.062)	0.017 (0.051)	0.014 (0.075)	0.027 (0.068)	0.036 (0.085)	0.041 (0.075)
IVW$_{LL}$	135.187 (444.534)	309.806 (> 999)	184.623 (> 999)	142.048 (896.464)	158.702 (801.631)	519.801 (> 999)	> 999 (> 999)	> 999 (> 999)

IV, instrumental variable; IVW$_{LI}$, inverse-variance weighted estimator with linear construction; IVW$_{LL}$, inverse-variance weighted estimator with non-linear construction; LIML, limited information maximum likelihood; 2SLS, two-stage least square; 2SPS$_{PR}$, two-stage predictor substitution with probit link; 2SPS$_{LL}$, two-stage predictor substitution with log link; 2SRI$_{PR}$, two-stage residual inclusion with probit link; 2SRI$_{LL}$, two-stage residual inclusion with log link.

The sample size n is 100,000, and the simulation is iterated 200 times. The true causal risk differences is approximately 0.173. The mean and empirical standard error (ESE) represented as "mean (ESE)" in the table are summarized. The analyses utilized 50 out of the true 500 SNPs. For the WIV methods, the datasets were split into two subsets, each containing 50,000 samples.

[b] ρ is a correlation coefficient between two unmeasured confounders.
